# Comparing methods for statistical inference with model uncertainty

**DOI:** 10.1073/pnas.2120737119

**Published:** 2022-04-11

**Authors:** Anupreet Porwal, Adrian E. Raftery

**Affiliations:** ^a^Department of Statistics, University of Washington, Seattle, WA 98195;; ^b^Department of Sociology, University of Washington, Seattle, WA 98195

**Keywords:** Bayesian model averaging, interval estimation, LASSO, model selection, parameter estimation

## Abstract

Probability models are used for many statistical tasks, notably parameter estimation, interval estimation, inference about model parameters, point prediction, and interval prediction. Thus, choosing a statistical model and accounting for uncertainty about this choice are important parts of the scientific process. Here we focus on one such choice, that of variables to include in a linear regression model. Many methods have been proposed, including Bayesian and penalized likelihood methods, and it is unclear which one to use. We compared 21 of the most popular methods by carrying out an extensive set of simulation studies based closely on real datasets that span a range of situations encountered in practical data analysis. Three adaptive Bayesian model averaging (BMA) methods performed best across all statistical tasks. These used adaptive versions of Zellner’s *g*-prior for the parameters, where the prior variance parameter *g* is a function of sample size or is estimated from the data. We found that for BMA methods implemented with Markov chain Monte Carlo, 10,000 iterations were enough. Computationally, we found two of the three best methods (BMA with $g=\sqrt{n}$ and empirical Bayes-local) to be competitive with the least absolute shrinkage and selection operator (LASSO), which is often preferred as a variable selection technique because of its computational efficiency. BMA performed better than Bayesian model selection (in which just one model is selected).

## Significance

Choosing a statistical model and accounting for uncertainty about this choice are important parts of the scientific process and are required for common statistical tasks such as parameter estimation, interval estimation, statistical inference, point prediction, and interval prediction. A canonical example is the choice of variables in a linear regression model. Many ways of doing this have been proposed, including Bayesian and penalized regression methods, and it is not clear which are best. We compare 21 popular methods via an extensive simulation study based on a wide range of real datasets. We found that three adaptive Bayesian model averaging methods performed best across all the statistical tasks and that two of these were also among the most computationally efficient.

Statistical analysis is often carried out using probability models for the data at hand. In this context, five of the most important statistical tasks are parameter estimation, interval estimation, inference about model parameters, point prediction, and producing prediction intervals.

These tasks often have to be carried out in the context of model uncertainty, where several different statistical models are plausible. One canonical example is variable selection in linear regression, where a set of candidate variables is considered, and all possible subsets of these candidate variables define possible models. Consider the linear regression model:

$$Y=\alpha 1_{n}+X\beta +\epsilon \;\epsilon ∼N(0,\sigma^{2}I),$$

where $Y\in R^{n}$ is a response variable of interest, and $X=(X_{1},\ldots ,X_{p})\in R^{n\times p}$ is a set of *p* possible covariates, *α* is the scalar intercept, and β is the p×1 vector of regression coefficients. For concreteness, we focus on this example here. Other examples include the choice of functional forms of the variables and the choice of error distribution, for instance, to account for potential outliers.

Many methods have been proposed for statistical analysis using linear regression models in the presence of model uncertainty. When the model is known in advance and only its parameters have to be estimated, there is consensus on how to do statistical analysis using it, using either a frequentist or Bayesian approach. When the model is to be determined as part of the analysis, however, things are less clear, and the large number of competing approaches can leave it unclear how to proceed. Here we compare 21 of the most prominent methods.

Historically, one approach has been to determine the variables in a model subjectively using subject matter expertise, but this often leaves open questions, and a data-based approach is desired for at least some of the variables. Another approach is to always include all the candidate variables, but this can lead to poor statistical performance when there are many such variables. Many of the early statistical approaches were stepwise methods, in which variables were sequentially added or removed on the basis of significant tests, but these have not been found to have good theoretical or empirical properties (1, 2).

In the past 30 y, many more satisfactory methods have been proposed. Most of these are either Bayesian techniques or penalized likelihood-based approaches.

Many of the Bayesian techniques are some form of Bayesian model averaging (BMA) (3–6); several reviews of the BMA literature are available (7–13). The basic idea of BMA is that the predictive distribution of a quantity of interest (either a parameter or an observable future quantity) is a weighted average of its predictive distributions under the different candidate models, where the weights are equal to the models’ posterior probabilities given the data at hand.

BMA has some good theoretical properties (14). BMA point estimators and predictions minimize mean squared error; BMA estimation and prediction intervals are calibrated, and BMA predictive distributions have optimal performance in the log score sense (6). These properties hold on average over the prior distribution, extending similar results for Bayesian estimation (15), but the results are somewhat robust to this assumption (16). Used in this way, as a distribution of parameter values over which performance is averaged, the prior distribution has been referred to as the world distribution (17), the practical distribution (14), or the effect-size distribution (18), and analysis using this concept has been called empirical frequentist*.

The implementation of BMA involves several choices by the user, including the prior distribution of the model parameters under each model and the prior model probabilities. Also, the number of candidate models can be too large for them all to be feasibly evaluated. For example, the number of possible subsets of *p* regression variables is $2^{p}$; for *p* much beyond 25 or 30 this can be computationally prohibitive. Thus, the choice of analytic or computational approximations must also be made. Together these choices lead to many possible implementations of BMA.

For the parameter prior distribution in linear regression, several default choices have been proposed. Among the first was the Zellner–Siow Cauchy prior, with a standard Jeffreys prior for the intercept and error variance (17, 19). We treat this as a reference method and call it the Jeffreys–Zellner–Siow (JZS) prior.

Another early prior was the Zellner *g*-prior (20). Consider a binary vector $\gamma =(\gamma_{1},\gamma_{2},\ldots ,\gamma_{p})$ that indicates which explanatory variables are part of model $M_{\gamma }$, so that $\gamma_{j}=1$ if the variable *X_j_* is present in $M_{\gamma }$ and 0 if not. We use Zellner’s *g*-prior in the form

$$\begin{array}{l} \pi_{\gamma }(\beta_{\gamma }|\alpha ,\sigma^{2},g)∼N(\beta_{\gamma }|0,g\sigma^{2}{\left(X_{\gamma }^{T}X_{\gamma }\right)}^{-1}),\\ \pi_{\gamma }(\alpha ,\sigma )\propto \sigma^{-1}, \end{array}$$

where N denotes the multivariate normal distribution, and $X_{\gamma }$ is the $n\times p_{\gamma }$ matrix consisting of the covariates *X_j_* for which $\gamma_{j}=1$ (9). The prior variance of the regression parameters is controlled by the user-specified value *g*, and the effective prior sample size is *n* / *g*, where *n* is the sample size.

Various choices of *g* have been proposed (13). Zellner proposed using *g* = *n*, corresponding to a prior sample size of 1; this has been called the unit information prior (UIP) (21). Another choice is *g* = 1, corresponding to a prior sample size of *n* (22), one justification being that studies have sample sizes designed to have the power to detect effects of known sizes, so that the prior and sampling variances are similar. An intermediate choice is $g=\sqrt{n}$ (9), with a prior sample size of $\sqrt{n}$; this has been found to work well in high-dimensional settings (23). The benchmark prior where $g=\max\{n,p^{2}\}$ has also been recommended (9); it combines the consistency properties of the UIP with the good small sample performance of the risk inflation factor (RIC) (24).

The UIP can also be approximated by the Bayesian information criterion (BIC) (25, 26). The Akaike information criterion (AIC) can be used as the basis for an approximation to the posterior model probabilities under a prior that is similar to Zellner’s *g*-prior with *g* = 1, i.e., with an equivalent prior sample size of *n* (27, 28).

An alternative is not to use a specified *g* but instead to estimate *g* from the data. This can be done in an empirical Bayes way, either for each model separately (29) or globally (30, 31). It can also be done in a more fully Bayesian way, by specifying a prior on *g*, such as the hyper-g approach (32).

A different type of prior used in BMA is the nonlocal prior (NLP) (33, 34), which removes mass close to zero. The horseshoe (35) is a Bayesian method but not a BMA method, with a prior that favors sparsity. The spike and slab method approximates the zero values of lower-dimensional models with continuous distributions around zero (5, 36).

In the frequentist setting, penalized likelihood approaches convert the variable selection problem into an optimization problem. The function to be optimized usually involves the squared error loss function with a penalty term $h_{\lambda }(\beta )$ on the coefficients β, in which case

[1] $$\begin{array}{l} \hat{\beta }=\underset{\beta \in R^{p}}{\arg\;\min}{\left(Y-\alpha 1_{n}-X\beta \right)}^{T}\\ \;(Y-\alpha 1_{n}-X\beta )+h_{\lambda }(\beta ). \end{array}$$

The estimates from these techniques can also be viewed as maximum a posteriori (MAP) estimates under a prior of the form $p(\beta )\propto \exp\;\{-h_{\lambda }(\beta )\}$. The least absolute shrinkage and selection operator (LASSO) (37) was the first and remains perhaps the most widely used technique in this class, where the penalty takes the form $h_{\lambda }(\beta )=\lambda \sum_{j=1}^{p}|\beta_{j}|$ and constrains the *l*_1_ norm of the parameter vector. The popularity of the LASSO is due to factors that include the computational efficiency of the least angle regression and coordinate ascent algorithms that can be used to estimate it (38, 39); its ability to provide a sparse estimate of β; and the oracle property, namely, that the LASSO will asymptotically find a superset of the correct predictors (40).

However, LASSO also suffers from several known issues. The oracle property ensures only that the true predictors will be asymptotically part of the selected model but not the converse, so that there can be many false positive selections, even asymptotically. LASSO also tends to overshrink the true signals in the observed data and hence produce biased estimates (41). It can also be unstable in the presence of highly correlated covariates. As pointed out by Holmes (ref. 42, p. 280), “In the presence of strong correlations between predictors with differing effect sizes, frequentist sparsity approaches, including the lasso, will tend to select a single variable within a group of collinear predictors, discarding the others in the pursuit of sparsity. However, the choice of the particular predictor might be highly variable and by selecting one we may ignore weak (but important) predictors which are highly correlated with stronger predictors.”

The LASSO has a constant rate of penalization for all coefficients which can cause excessive shrinking of nonzero components. Some of the other popular penalty methods vary in the shape or rate of penalty applied to the coefficients. The smoothly clipped absolute deviation (SCAD) (43) and minimax concave penalty (MCP) (44) methods involve a nonconvex penalty which is constant for smaller coefficients and decreases to 0 for larger coefficients. The elastic net (45) involves a convex combination of ridge and LASSO penalties, encouraging grouping effects among strongly correlated variables, and thus addresses one concern mentioned above for the LASSO. Like the LASSO, these methods threshold some coefficients to zero, leading to simultaneous variable selection and estimation.

A common issue with penalized likelihood approaches is the lack of uncertainty quantification since variable selection is an outcome of the constrained optimization problem and not a probabilistic statement of inclusion (46–48). As a result, the zeros induced may not be the same zeros that one would get from a full variable selection approach (49). They also do not provide a way to account for model uncertainty. Expectation–maximization variable selection (EMVS) (50) and the spike-and-slab LASSO (SS LASSO) (51) are two methods that synthesize ideas from BMA and penalized likelihood. In principle, they could quantify uncertainty, but that has not yet been implemented in the associated software.

It is not clear which of the many proposed methods to use. Among penalized likelihood methods, LASSO probably remains the most used, perhaps because it was the first one proposed, there is a well-defined software package to implement it (the glmnet R package), and it is fast (52). Among Bayesian methods there is less clarity, and the relative performance of Bayesian and penalized likelihood methods is also not clear.

**Fig. 1.**
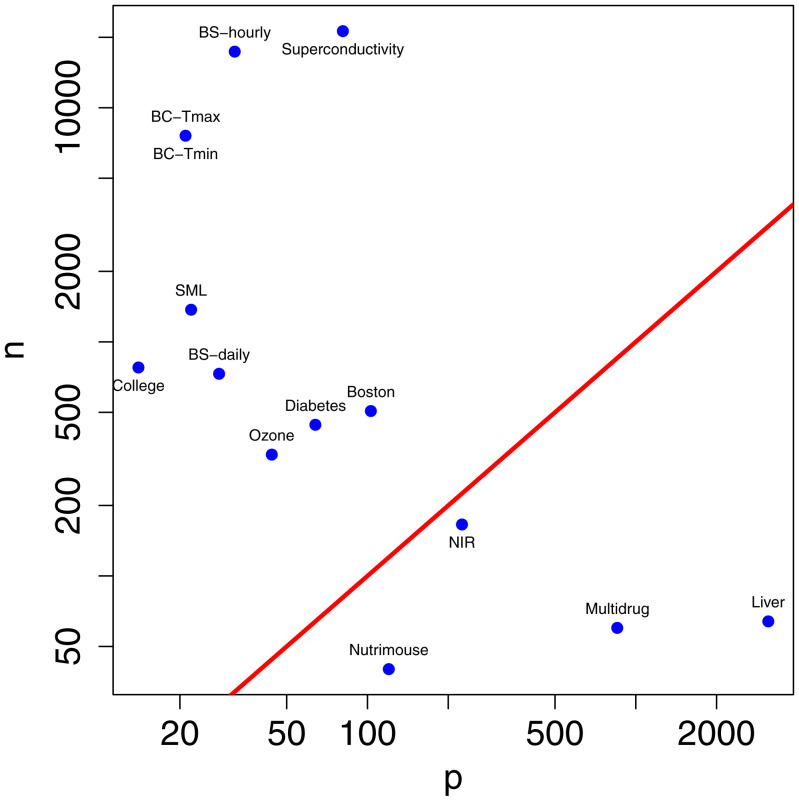
Sample size *n* versus the number of candidate variables *p* for the 14 datasets on which our simulation studies are based. The *n* = *p* line is shown in red.

To clarify this, we carried out an extensive set of simulation studies based closely on real datasets that span a range of situations encountered in practical data analysis. This is in contrast with many simulation studies in the statistical literature whose design is determined by the investigators without direct reference to data. The simulation design, the metrics, and the underlying datasets are described in *Materials and Methods*. Fig. 1 shows the sample size and the number of candidate variables for the different datasets. These include classic statistical situations where the sample size is much larger than the number of variables, high-dimensional situations where the number of variables exceeds the sample size, and intermediate situations where the two are of the same order of magnitude.

## Results

**Fig. 2.**
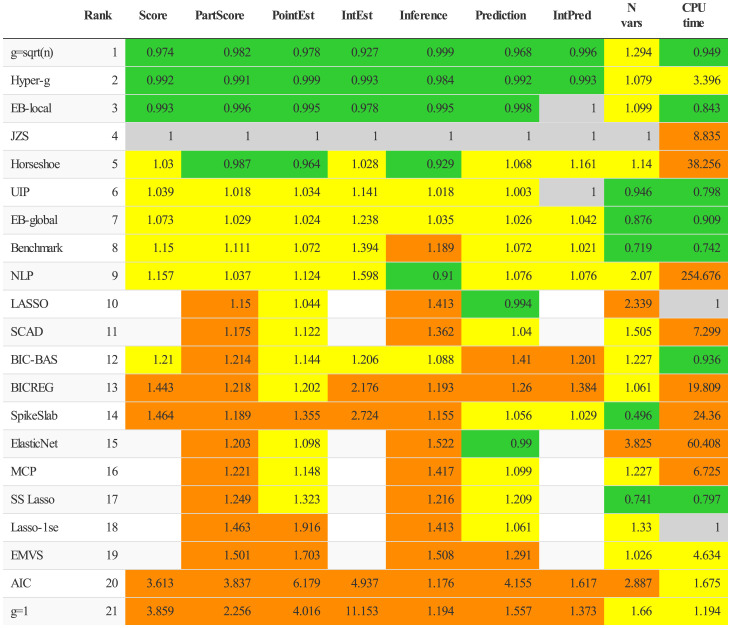
Performance of 21 methods for inference in linear regression under model uncertainty: “PointEst” is the RMSE for point estimation, “IntEst” is the MIS for interval estimation, “Inference” is 1 – the AUPRC, “Prediction” is the RMSE for point prediction, and “IntPred” is the MIS for interval prediction. “N vars” is the average number of variables used for the task. All metrics are standardized to equal 1 for the JZS method. See *Results* and *Materials and Methods* for more information about the ranking and coloring and the definitions of the methods and metrics. Note that BICREG denotes the BICREG-SIS method, in which sure independence screening is used first to reduce the number of variables to 30.

The results are shown in Fig. 2. Performance metrics are shown for all 21 methods for each of point estimation, interval estimation, inference, prediction, and interval prediction. All metrics are relative to the score for the JZS method, taken as the reference, and averaged across datasets. Detailed results of performance metrics for the simulation studies based on each of the 14 datasets can be found in *SI Appendix*. The score column shows the average of the five metrics for each method. For seven of the methods, interval estimation and interval prediction metrics were not available as the methods did not provide uncertainty assessments, and so we calculated the “PartScore,” which is the average of the three remaining metrics. In all cases, a lower score is better.

We first ranked the methods according to Score. We then ranked the methods for which Score was not available according to PartScore, ranking each one as highly as possible without changing the Score order. Results are colored green if the method performed better than the reference JZS method, while they are colored red if there was a substantial gap between them and the best methods. Yellow indicated that the method did not perform as well as the reference method but was not substantially worse than competing methods either. We also showed the average number of variables used and the central processing unit (CPU) time. For CPU time, LASSO was taken as the reference as it has generally been viewed as a computationally efficient method.

Overall, the ranking of the methods was similar from the different metrics. Strikingly, the venerable JZS method, now in its fifth decade, performed well and was competitive with all other methods, except that it required more CPU time than many. The top scoring methods were three adaptive *g*-prior methods: $g=\sqrt{n}$, the hyper-g method, and the local empirical Bayes method, which were the only methods to consistently outperform the reference method. Other Bayesian methods with nonadaptive priors rounded out the top eight spots. Interestingly, *g* = 1 and AIC were the worst performing methods. An advantage of the Bayesian methods is that they organically yield uncertainty statements, unlike the penalized likelihood methods.

LASSO was the top penalized likelihood method, doing particularly well for point prediction, as did the Elastic Net—comparable to the top Bayesian methods for this task, although not for the other tasks. However, they both selected far more variables on average than the Bayesian methods—twice as many or more in most cases without any noticeable increase in predictive performance. Plots of prediction accuracy, given by *R*^2^, versus average model size, denoted by $\hat{p}$, for all datasets are available in *SI Appendix*.

A surprise was that two of the top three methods were efficient computationally even though they were Bayesian, comparable to LASSO despite the reputation of Bayesian methods for being slow. This is partly because we used a default of 10,000 Markov chain Monte Carlo (MCMC) iterations, which is far fewer than the default in the BAS R package used to implement these methods (53). This clearly gave adequate performance. Performance might be improved slightly with more iterations but at the cost of computational efficiency. The hyper-g method is substantially slower, which seems to be due to its greater complexity, but this may be a worthwhile tradeoff given its good performance. Several of the other methods were extremely slow. One needs to be cautious in interpreting the CPU time results as they reflect the coding efficiency of the implementations as well as the intrinsic computational efficiency of the methods. For most methods we used the developers’ packages with default settings, and these could clearly often be sped up.

One question is whether inferences are sensitive to the choice of model selection/model averaging method. To provide a partial answer, we compared the results for our 14 datasets for the top three methods identified by our study. Scatterplots of parameter estimates and posterior inclusion probabilities are shown in *SI Appendix* for all 14 datasets. We found that the (model-averaged) parameter estimates were very similar between the three methods for the 10 tall datasets (with *p*  <  *n*) and less similar but still highly correlated for the four wide datasets (with *p*  >  *n*). The posterior inclusion probabilities were similar between methods for the tall datasets but less so for the wide datasets. The $g=\sqrt{n}$ method tended to favor models with slightly more variables than the hyper-g and Empirical Bayes (EB)–local methods.

### Comparison of BMA with Bayesian Model Selection.

An alternative to BMA is Bayesian model selection (BMS), in which just one model is selected. When several candidate models are available, a researcher can choose to select one model or perform model averaging. BMS refers to selection of one model from a list of candidate models based on the data (7, 10). One choice for BMS is to select the model with the highest posterior probability in model search, also known as the MAP model. We compared the performance of BMA and BMS for the top three methods identified in the previous section: $g=\sqrt{n}$, hyper-g, and EB-local.

We used the same performance metrics as before. As before, all metrics are relative to BMA under the JZS prior, except for computation time, for which LASSO was used as the reference. The results are shown in Table 1. The BMS versions of the top three methods performed worse than the corresponding BMA versions in terms of all the metrics.

**Table 1.**
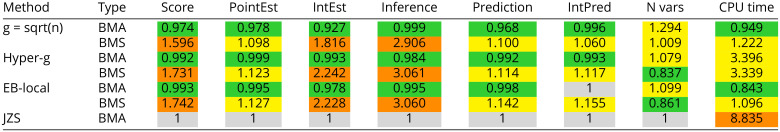
Comparison of BMA and BMS for top three methods

## Discussion

Several previous comparisons of existing methods have been carried out. They have tended to be based on a narrower range of methods than we consider here, to be based on simulation experiments whose connection to empirical data is less clear, and to base comparisons on a subset of the statistical tasks of interest.

Fernández et al. (9) did a simulation study based on a nonempirical design (54) and compared methods based on their ability to recover the true underlying model as the MAP model and assess predictive performance using log-predictive scores. Hence, their comparisons were based on only two statistical tasks, namely inference and point prediction. They considered only BMA methods. They found a UIP-based method with *g* = *n* to work best when $n<p^{2}$ and an RIC-based method (24) with $g=p^{2}$ to work best otherwise, but they pointed out that the RIC-based method is not model-selection consistent. We have included the resulting combined method in our study under the name “benchmark prior.” The only other method in their study that is also in ours is the $g=\sqrt{n}$ method, which they found to be outperformed by BIC, in contrast with our findings here.

Eicher et al. (55) considered the same BMA methods as in ref. 9, considered prediction for a well-known economic growth dataset and for several simulations with the same nonempirically based design, and again found BIC and UIP to do best. Our results here are based on a wider and more empirically based set of simulations, which may help explain the different results. Liang et al. (32) also carried out a nonempirically based simulation study using the design of Cui and George (56) and found the hyper-g prior to be competitive with other BMA methods in terms of parameter estimation, including several that we have considered here (but they did not include the $g=\sqrt{n}$ method).

Celeux et al. (57) carried out another nonempirical simulation study to assess quality of inference and assessed point prediction using two small real datasets; they assessed 15 methods, of which 7 were in common with ours. They focused on the situation where *p* is close to *n*. Like us, they found Bayesian methods to outperform non-Bayesian ones.

Deckers et al. (58) compared a subset of the Bayesian techniques discussed in our study, specifically the UIP and RIC, or benchmark prior, and LASSO with multiple testing procedures (MTPs) controlling false discovery rate. They focused their comparison on the model inference performance of these procedures using size-adjusted power, i.e., comparison of power (number of correctly selected variables) in situations where procedures infer similar size models. In their comparison over a nonempirical simulation study, they found that BMA was slightly more powerful given the size than the MTPs and LASSO. Their comparison did not focus on other statistical inference tasks of prediction and estimation.

Bhadra et al. (59) compared variants of the horseshoe, LASSO, and SCAD in terms of their performance in variable selection, using the nonempirically based simulation design of Zhao and Yu (60). They found the horseshoe to do best, then SCAD, both substantially dominating LASSO. This agrees with our ranking in terms of inference from Fig. 2, but we found LASSO to overtake SCAD when other statistical tasks were also taken into account. Forte et al. (13) compared different BMA software packages in terms of computational performance and found the BAS package (53) to dominate others in terms of speed, as we also found. However, they also warned against the use of the MCMC + BAS method within BAS, which they reported does not provide reliable estimates of the inclusion probabilities, and instead recommended the method MCMC. They also commented on the very high memory demands of BAS. Here we used their recommended method MCMC and found it to work well.

One can also evaluate methods in terms of theoretical properties. One is model-selection consistency (9), which says that if the true model is among the candidate models considered, the method will select it with probability approaching 1 as the sample size increases indefinitely. All three of our top-ranked methods satisfy this unless the true model is the null model with no predictors (9, 32). However, LASSO does not have this property (60).

A second property is whether the method is subject to Bartlett’s paradox (61), according to which if the data are held fixed and the prior variance increases without bound, then BMA will select the null model with probability tending to 1, regardless of the data. None of our top three methods is subject to this as they do not allow the prior variance to increase without bound.

A third consideration is whether the method is subject to the so-called “information paradox” (32). This arises when, for fixed *n* and *p*, the data provide maximal support for a larger model, for example, when $R^{2}\to 1$. One could argue that in this case, the Bayes factor for this model against any submodel should tend to infinity with the sample size. However, *g*-priors with fixed *g* do not have this property, and indeed in that case the Bayes factor has a finite (although usually very high) upper bound. It has been argued that this is undesirable, making them subject to the information paradox. The hyper-g and EB-local methods are not subject to this, but the $g=\sqrt{n}$ prior is, which could be argued to be a disadvantage of the latter.

However, one might question the relevance of the information paradox to the choice of method (62). If $R^{2}=1$ when *n* is small, this will often be because of the inherent discreteness of most data, which are rarely measured or recorded with full precision but rather to within a certain measurement tolerance (for example, a certain number of significant digits). In that case, the fact that the Bayes factor for an additional variable is bounded above could be viewed as an advantage. The linear regression model models the observed response variable as a continuous variable, thus measured with infinite precision. This is actually an approximation, which is usually inconsequential, but is relevant for assessing the relevance of the information paradox. If the discreteness of observed data were accounted for in the model, the information paradox would never arise.

For example, the famous data on heights of fathers and sons in England (63, 64) are reported to the nearest inch. If one took a sample of size 3 from these data, say (father, son) = (62.5, 64.5), (67.5, 69.5), (70.5, 72.5), and regressed son’s height on father’s height, one would find that $R^{2}=1$ and the standard *F* statistic is infinite. In this case, one would not want the Bayes factor for the effect of father’s height to be infinite, but it is infinite for the hyper-g and EB-local priors, while for the $g=\sqrt{n}$ prior it is 1.65. The latter represents positive but weak evidence for an effect, which seems more reasonable than an infinite Bayes factor corresponding to absolute certainty based on three data points.

Beyond that, the upper bound on the Bayes factor is typically very high for even moderate *n*. For example, for *n* as low as 20, it is over 4 million. So even if the existence of an upper bound on the Bayes factor were to be viewed as undesirable, it would have no practical effect. Overall, this suggests that the information paradox may not be a disadvantage for the $g=\sqrt{n}$ prior and others that it affects and may even be a positive feature.

We have focused here on the choice of prior distribution for model parameters. BMA also requires a prior on the models themselves, and we have used default choices: either a uniform prior over all models or a uniform prior on model size. It would be worth carrying out a similar analysis to the present one to compare different possible model priors.

Given the good performance of the $g=\sqrt{n}$ prior of ref.  9, it is of interest how it relates to the popular BIC criterion, which corresponds approximately to *g* = *n* and performed less well in our experiments. Let us consider just two models: the null model and a regression model of interest, with *d* variables. Then if *B* is the Bayes factor for the regression model against the null model, the exact result is $-2\log\;B=(n-1)\log\;\{1+\sqrt{n}(1-R^{2})\}-(n-1-d)\log\;(1+\sqrt{n}).$ The BIC approximation is $-2\log\;B\approx n\log\;(1-R^{2})+d\log\;(n)$. A similar approximation with the $g=\sqrt{n}$ prior is $-2\log\;B\approx n\log\;(1-R^{2})+d(\log\;(n)/2)+\sqrt{n}(1-R^{2})R^{2}$. The last term does not involve the number of parameters directly, and so the complexity penalty in the Bayes factor with the $g=\sqrt{n}$ prior is effectively half that in the BIC.

We have focused on one specific type of model uncertainty in one statistical setting, namely, uncertainty about which variables to include in a linear regression model. This has been much studied and arises frequently in science, as well as being a canonical example for other statistical models. However, there are many other statistical settings in which the same issue arises, and it would be of interest to carry out similar comparative studies. In linear regression itself, there are the choices of error distribution and functional form of the variables. The same issues arise in generalized linear models such as logistic regression and Poisson regression, in addition to the choice of link function and mean-variance relationship. Similar model choice issues arise with Bayesian hierarchical models and many other model classes. We expect that our main conclusion, that BMA with an adaptive parameter prior performs well, would carry over to other settings.

## Materials and Methods

### Statistical Methods for Comparison.

The 21 methods we compare are listed in Table 2, along with references, the R package used, and the function call used. All the *g*-prior methods implemented using the BAS package, and the NLP methods implemented using the mombf package, use the beta-binomial (1, 1) prior as the default model space prior. For high-dimensional datasets with *p*  >  *n*, a truncated beta-binomial (1, 1) prior is used as the model space prior; this assigns probability zero to any model with size greater than *n* – 2. The BICREG-SIS method assumes a uniform prior over the model space. For methods implemented using the BAS package, a combination of the Metropolis–Hastings algorithm, as in the MCMC model composition algorithm (54), with a random swap between a currently included and a currently excluded variable, is used for model space exploration.

**Table 2.** Variable selection methods compared in this study

Method	Authors	Implementation (R package–version)	Function
$g=\sqrt{n}$	Fernández et al. (9)	BAS-V1.5.5 (53)	bas.lm(…, prior=”g-prior”, alpha = sqrt(n))
Hyper-g	Liang et al. (32)	BAS-V1.5.5 (53)	bas.lm(…, prior=”hyper-g”)
EB-local	Hansen and Yu (29)	BAS-V1.5.5 (53)	bas.lm(…, prior=”EB-local”)
JZS	Zellner and Siow (19)	BAS-V1.5.5 (53)	bas.lm(…, prior=”JZS”)
Horseshoe	Carvalho et al. (35)	horseshoe-V0.2.0 (65)	horseshoe()
UIP	Kass and Wasserman (21)	BAS-V1.5.5 (53)	bas.lm(…, prior=”g-prior”, alpha = n)
EB-global	Clyde and George (30) and George and Foster (31)	BAS-V1.5.5 (53)	bas.lm(…, prior=”EB-global”)
Benchmark	Fernández et al. (9)	BAS-V1.5.5 (53)	bas.lm(…, prior=”g-prior”, alpha = max(n,*p*^2^))
NLP	Rossell and Telesca (34) and Johnson and Rossell (33)	mombf-V2.2.9 (66)	modelSelection()
LASSO*	Tibshirani (37)	glmnet-V3.0.2 (52)	cv.glmnet()
SCAD	Fan and Li (43)	ncvreg-V3.11.2 (67)	cv.ncvreg(…, penalty=”SCAD”)
BIC-BAS	George and Foster (31)	BAS-V1.5.5 (53)	bas.lm(…, prior=”BIC”)
BICREG-SIS	Raftery (26) and Fan and Lv (68)	BMA-V3.18.12 (69)	bicreg()
Spike slab	George and McCulloch (36)	BoomSpikeSlab-V1.2.3 (70)	lm.spike()
Elastic net	Zou and Hastie (45)	glmnet-V3.0.2 (52)	cv.glmnet(, alpha)
MCP	Zhang et al. (44)	ncvreg-V3.11.2 (67)	cv.ncvreg(…, penalty=”MCP”)
SS lasso	Ročková and George (51)	SSLASSO-V1.2.2 (51)	SSLASSO()
EMVS	Ročková and George (50)	EMVS-V1.1 (71)	EMVS()
AIC	George and Foster (31)	BAS-V1.5.5 (53)	bas.lm(…, prior=”AIC”)
*g* = 1	van Zwet (22)	BAS-V1.5.5 (53)	bas.lm(…, prior=”g-prior”, alpha = 1)

^*^LASSO-1se has the same reference as LASSO.

### Datasets.

We carried out 14 simulation studies, each one based on a different publicly available real dataset, from a variety of fields including social sciences, healthcare, genome sciences, physical sciences, chemistry, and engineering (Table 3). We selected several of our datasets by filtering all the datasets in the University of California, Irvine, machine learning repository as follows. We filtered datasets with default task as regression, attribute type as numerical, data type as multivariate/univariate, and number of attributes between 10 and 100. We further restricted our attention to datasets with *p*  >  20 and *n*  <  25,000. This reduced our list of UCI datasets to four: the bias correction, bike sharing, SML, and superconductivity datasets. Note that the bias correction and bike sharing datasets each have two versions based on choice of outcome and frequency.

**Table 3.** Datasets used in the study

Dataset name	Sample size (N)	Covariates (p)	Source
College	777	14	ISLR (72)
Bias Correction-Tmax	7,590	21	UCI ML repository
Bias Correction-Tmin	7,590	21	UCI ML repository
SML2010	1,373	22	UCI ML repository
Bike sharing-daily	731	28	UCI ML repository
Bike sharing-hourly	17,379	32	UCI ML repository
Superconductivity	21,263	81	UCI ML repository
Diabetes	442	64	spikeslab (73)
Ozone	330	44	gss (74)
Boston housing	506	103	mlbench (75)
NIR	166	225	chemometrics (76)
Nutrimouse	40	120	mixOmics (77)
Multidrug	60	853	mixOmics (77)
Liver toxicity	64	3,116	mixOmics (77)

We also included several datasets that have been used as examples in the literature. We included the college dataset (78) as an example dataset where full enumeration of models is feasible. We included the diabetes (38) and ozone (1, 32) datasets and the Boston housing dataset with squares and interaction terms between its covariates. Finally, we included four high-dimensional datasets from chemometrics and genomics from the mixOmics (77) and chemometrics R packages (76). For all the datasets, the continuous predictors were standardized to have mean zero and variance 1, and the response variable was centered to have mean zero. The 14 datasets used in the simulation study are listed in Table 3. Details of dataset preprocessing are given in *SI Appendix*.

### Determining the Generating Model for the Simulation Study.

For our simulation study, we require a data generating model based on each of our real datasets. For datasets for which *p*  <  30, we performed all subsets regression using the leaps package in R (79) and selected the largest model with all variables significant at 0.05 level. For datasets with *p*  >  30, all subsets regression can be computationally intensive, and so we performed iterative sure independence screening (ISIS) (68) to reduce the number of variables. If the filtered list contained more than 30 variables, we further selected the top 30 variables with the highest *R*^2^ values under univariate regression. We then applied all subsets regression to the filtered list of covariates with the above criteria to find the data generating model for our simulation study.

Consider the Boston housing dataset (*n* = 506, *p* = 103) as an example. This includes 14 geographic housing variables, plus interactions and squares for each continuous variable, leading to 103 possible predictors. All subsets regression is not computationally feasible, so instead we used ISIS to get a filtered list of 81 variables. We then performed univariate regressions for each of the filtered variables to select the top 30 variables with the highest *R*^2^ values. Finally, we performed all subsets regression using the screened variables to get our data generating model with 23 variables and an *R*^2^ of 0.86.

### Simulation Design.

For each dataset, we chose a data generating model as described above to closely approximate the data. Using this model, we used the parametric bootstrap to generate 100 bootstrapped datasets with the same design matrix ***X*** but different simulated response vectors. We compared the performance of the different techniques for parameter estimation, interval estimation, and variable selection on these datasets for our simulation study using the metrics described below.

To evaluate the predictive performance of the methods, we divided each of the simulated datasets into 100 random 75–25% train–test splits. We trained the methods on the training data and used the test data to assess the predictive performance using the metrics described below. We calculated point predictions for each of the methods and posterior predictive intervals for Bayesian techniques that allow for uncertainty quantification.

We used the following metrics to compare the methods.

#### PointEst.

For point estimation, we calculated the root mean squared error (RMSE) of the parameter estimates as follows:

[2] $$\mathrm{RMSE}=\sqrt{\frac{1}{p}\sum_{i=1}^{p}{\left(\beta_{i,DG}-{\hat{\beta }}_{i}\right)}^{2}},$$

where $\beta_{i,DG},i=1,\ldots ,p$ denote the coefficients in the data generating model, and ${\hat{\beta }}_{i},i=1,\ldots ,p$ denote the posterior means of the coefficients for the Bayesian techniques and the estimated optimal coefficients for penalized likelihood based approaches.

#### IntEst.

The interval score (IS) (80) provides a balance between the narrowness of the intervals and the accuracy of the coverage. It is a sum of two components: the first rewards narrow intervals, and the second rewards accurate coverage. For a variable *z*, the IS is given by

[3] $$\begin{array}{ll} MIS_{\alpha }(l,u,z)=(u-l)+ & \frac{2}{\alpha }(l-z)1\{z<l\}\\ & +\frac{2}{\alpha }(z-u)1\{u<z\}, \end{array}$$

where *l* and *u* denote the upper and lower bounds of the (1−α)×100% posterior intervals of *z*. In order to assess the quality of the interval estimation, we considered the mean interval score (MIS) for the coefficients and calculated the average MIS across coefficients for each of the datasets. We used α=0.05.

#### Inference.

To compare the performance of the techniques for identifying the appropriate variables, we calculated the area under the precision recall curve (AUPRC) for each of the techniques. This gives an overall assessment of model selection quality and does not require a threshold to be chosen for the posterior inclusion probability of a covariate.

For penalized likelihood based approaches, the AUPRC was obtained by varying the cross-validation parameter *λ* from close to 0 (no penalization) to *λ_max_*, defined as the smallest value of *λ* for which none of the variables is included in the model (81). For the horseshoe, the AUPRC was obtained by varying the credible set levels leading to different number of variables being selected by the method. We report Inference with (1 – AUPRC) as our metric, and a lower value is better.

#### Prediction.

In order to assess the accuracy of point prediction, we calculated $R_{\mathrm{test}}^{2}$ as follows:

[4] $$R_{\mathrm{test}}^{2}=1-\frac{\sum_{i\in \mathrm{test}}{\left(y_{i}-\hat{y_{i}}\right)}^{2}}{\sum_{i\in \mathrm{test}}{\left(y_{i}-{\bar{y}}_{\mathrm{train}}\right)}^{2}},$$

where $\left\{y_{i}:i\in \mathrm{test}\right\}$ denotes the response variable of the test set, ${\hat{y}}_{i}$ denotes the corresponding predictions, and ${\bar{y}}_{\mathrm{train}}$ denotes the mean of the response variable in the training set. Note that this quantity can be less than zero, if the predictions perform worse than the baseline ${\bar{y}}_{\mathrm{train}}$.

#### IntPred.

To assess the quality of the prediction intervals, we calculated the interval score using Eq. **3** for each of the test set observations. Here *l* and *u* represent the lower and upper bounds of the (1−α)×100% posterior predictive interval for the test observation. We calculated the MIS, averaging IS over test set observations for each of the train–test splits. A lower MIS corresponds to a better interval forecast.

#### N vars.

To report sparsity levels, we recorded the average model size for the BMA techniques and the number of nonzero estimated coefficients for the penalized likelihood based approaches. For the horseshoe, we calculated a 95% credible interval and checked whether 0 was included in it to arrive at the model size. We denote the average model size by $\hat{p}$.

#### CPU time.

We recorded the average computation time (in seconds) taken by each technique to fit the model for one bootstrapped dataset.

## Supplementary Material

Supplementary File

## Data Availability

Previously published data were used for this work (https://archive.ics.uci.edu/ml/index.php) (76, 77).

## Footnotes

^*^J. O. Berger, World Meeting of the International Society for Bayesian Analysis, June 28–July 2, 2021, online.
