# Supplementary material for: Comparing methods for statistical inference with model uncertainty
Source: Proc Natl Acad Sci U S A. 2022 Apr 11;119(16):e2120737119. doi: 10.1073/pnas.2120737119 (PMC9169744; doi:10.1073/pnas.2120737119)
Supplement: Supplementary File [file pnas.2120737119.sapp.pdf]

Comparing Methods for Statistical Inference with Model Uncertainty : Supporting Information

Anupreet Porwal and Adrian E. Raftery

1 Datasets Description

Here we present a brief description of the data and the transformations used in the analyses:

- `College`: We model log of applications received on statistics collected for 777 US colleges from the 1995 issue of US news and world report. We remove `Enroll, Accept` due to potential causal relationship with number of applications. We perform a log transformation on number of full time undergraduates (`F.undergrad`) and part-time graduates (`P.undergrad`).

- `Bias Correction`: This data is for the purpose of bias correction of next-day maximum and minimum air temperatures forecast of the LDAPS model operated by the Korea Meteorological Administration over Seoul, South Korea. The input data is largely composed of the LDAPS model's next-day forecast data, in-site maximum and minimum temperatures of present-day, and geographic auxiliary variables. There are two outputs (i.e. next-day maximum and minimum air temperatures) in this data. We excluded station and date variable from our modelling. We model square root of maximum and minimum air temperature as a function of available covariates. Maximum temperature was not used in modeling of minimum temperature and vice versa.

- `SML2010`: The following dataset contains forecasts of several weather parameters and actuators state collected at a solar-powered house, known as Small Medium Large System (SMLsystem). The data was sampled every minute, computing and uploading it smoothed with 15 minute means. The goal is to model indoor temperature as a function of the above parameters. We excluded time and data variables. We also excluded Enthalpic motor and Enthalpic motor turbo since there was no variability in these variables across observations. We treated day of week as a factor variable.

- `Bike sharing`: We consider both the hourly and daily version of Bike sharing dataset that contains data for count of rental bikes between years 2011 and 2012 in Capital bikeshare system with the corresponding weather and seasonal information. We remove `instant, dteday` variables as these variable are indexing variables. We also remove `casual, registered` variables since `count` is defined as sum of these two variables. We rescale the normalised versions of `temp, atemp, windspeed` and `hum` using normalization constants available in the read me file available on UCI. We treat `yr, weathersit, holiday, mnth, weekday` and `season` as a factor variable. We remove `workingday` as it is determined by `weekday` deterministically. The outcome variable for daily data is square root of `count`.

  For the hourly data, we group `hr` into 5 categories: `Latenight, EarlyMorning, Morning, Evening` and `Night`. The outcome variable for daily data is cube root of `count`.

- `Superconductivity`: We model the cube root of critical temperature based on based on the 81 features extracted from the superconductor's chemical formula. The sample size is 21,263.

- `Diabetes`: The data consists of 442 patients in which the response of interest is a quantitative measure of disease progression. The data includes 10 baseline measurements for each patient, in addition to 45 interactions and 9 quadratic terms, for a total of 64 variables for each patient.

- `Ozone`: The dataset consists of daily measurements of the maximum ozone concentration near Los Angeles and eight meteorological variables. We model log of ozone concentration using the eight meteorological variables, plus interactions and squares, leading to 44 possible predictors.

- `Boston Housing`: This dataset consists of housing data for 506 census tracts of Boston from the 1970 census. We examine median value of owner-occupied homes (in USD 1000's) using 13 base variables, their interactions and quadratic terms, for a total of 103 variables per observation.

- `NIR`: This dataset consists of 235 variables containing first derivatives of near infrared spectroscopy (NIR) absorbance values for 166 alcoholic fermentation mashes of different feedstock. The outcome variables is concentration of glucose (in g/L) in the feedstock.

- `nutrimouse`: The data set come from a nutrigenomic study in the mouse containing observations of 40 mice where hepatic fatty-acid concentrations are regressed upon the expression of 120 potentially relevant genes measured in liver cells. The response variable is `C16.0`.

- `multidrug`: The Multidrug data are from a pharmacogenomic study investigating the relationship between the drug concentration and expression of the adenosine triphosphate binding cassette transporter (ABC3A). The X matrix comprises observations of the activity of 853 drugs on 60 different human cell lines, expressed as the concentration at which each drug leads to a 50% inhibition of growth for each cell line. The y variable is the measured expression of ABC3A in each cell line.

- `liver.toxicity`: This dataset comes from a liver toxicity study containing the expression scores for 3116 genes in 64 rat subjects. We model the cholesterol concentration in the liver (in mg/dL) as a function of the expression scores of genes.

2 Scatter plots for estimated posterior inclusion probabilities (PIPs) and coefficients of top 3 methods for all datasets

Here are the scatter plots for PIPs and coefficients of top three methods. We observe that for tall datasets, Hyper-g and EBlocal agree and tend to select sparser models compared

to g=sqrt(n). For wide datasets, there is no agreement between methods on estimated coefficients and PIPs possibly due to presence of several highly correlated variables.

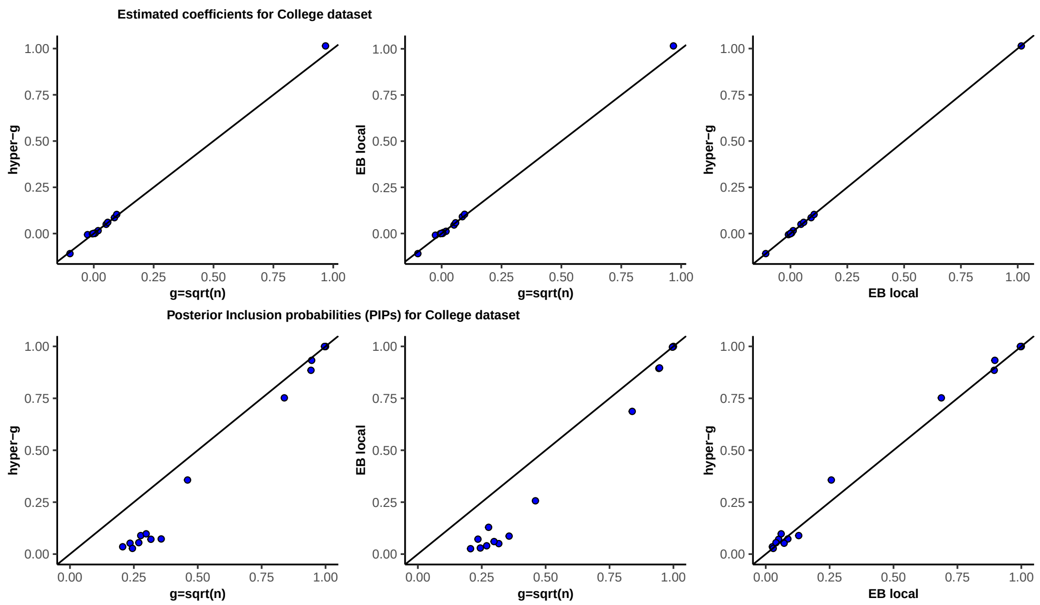

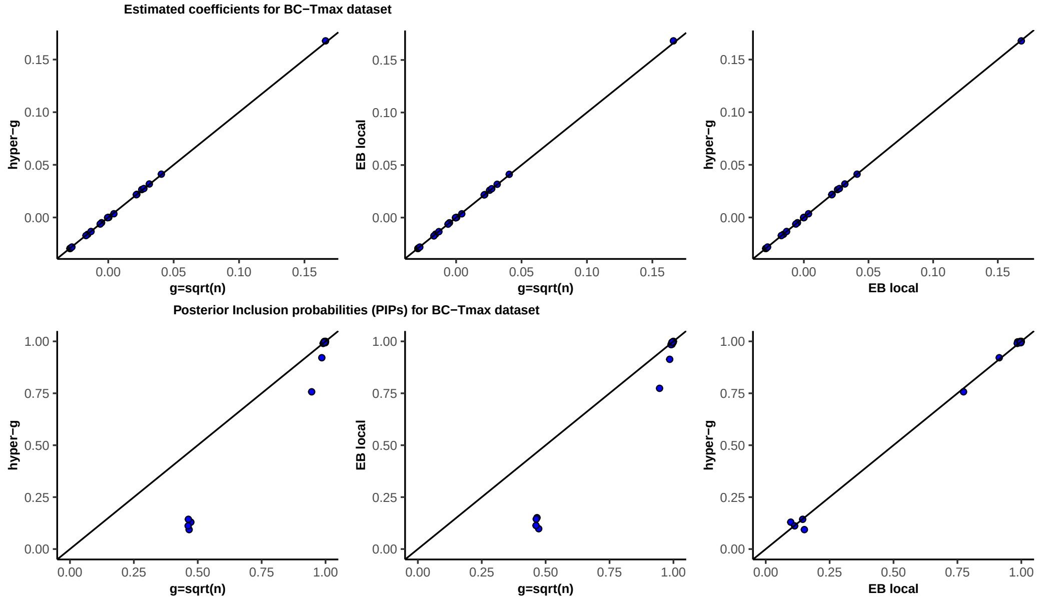
Estimated coefficients for BC−Tmax dataset
hyper−g
EB local
g=sqrt(n)
EB local
Posterior Inclusion probabilities (PIPs) for BC−Tmax dataset

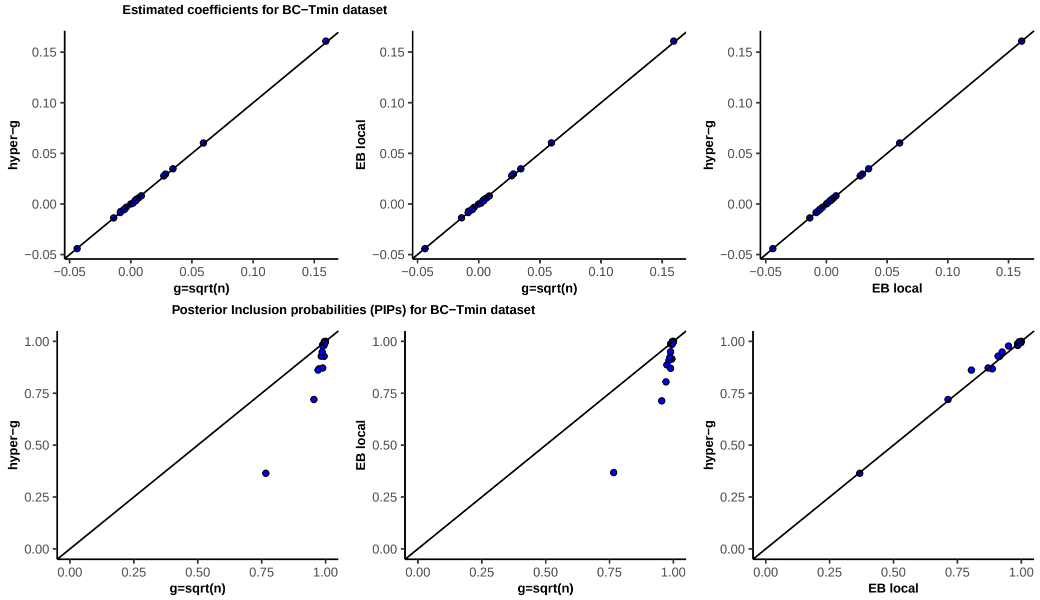
Estimated coefficients for BC−Tmin dataset
hyper−g
EB local
g=sqrt(n)
EB local
Posterior Inclusion probabilities (PIPs) for BC−Tmin dataset

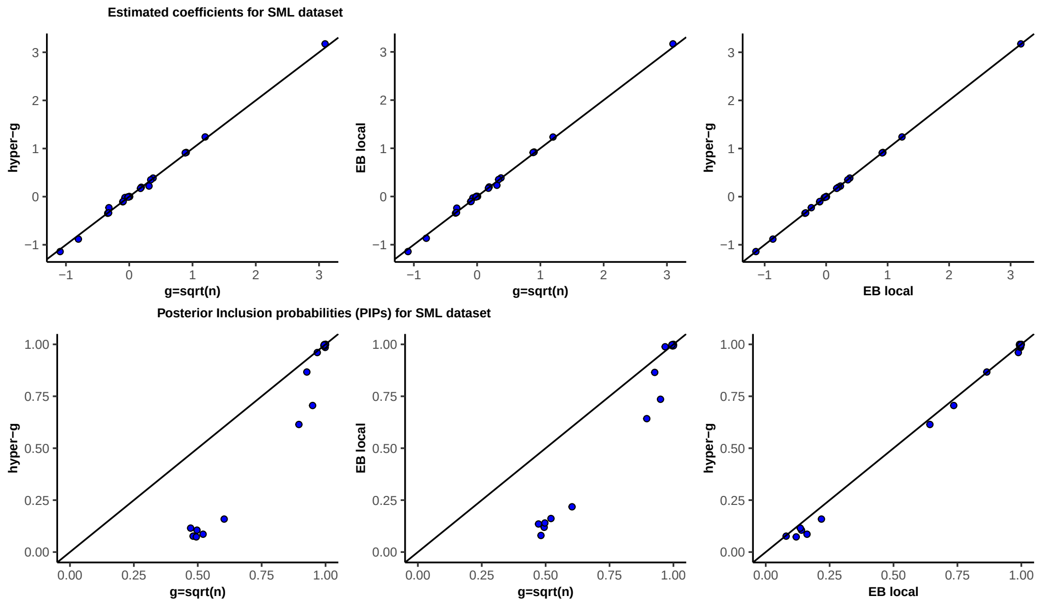
Estimated coefficients for SML dataset
hyper−g
EB local
g=sqrt(n)
EB local
Posterior Inclusion probabilities (PIPs) for SML dataset
hyper−g
EB local
g=sqrt(n)
EB local

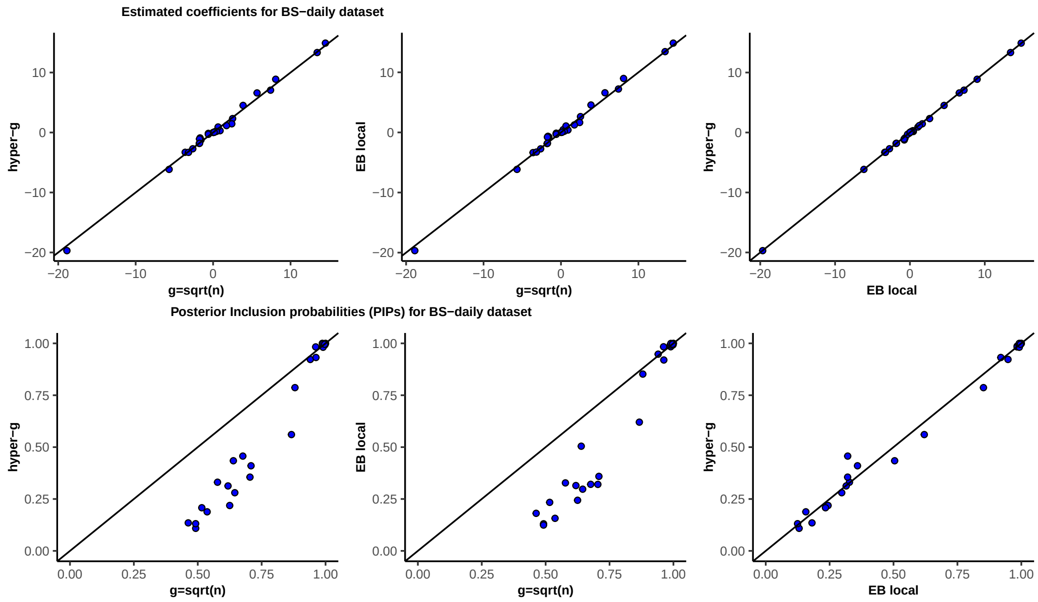
Estimated coefficients for BS−daily dataset
hyper−g
EB local
g=sqrt(n)
EB local
Posterior Inclusion probabilities (PIPs) for BS−daily dataset
hyper−g
EB local
g=sqrt(n)
EB local

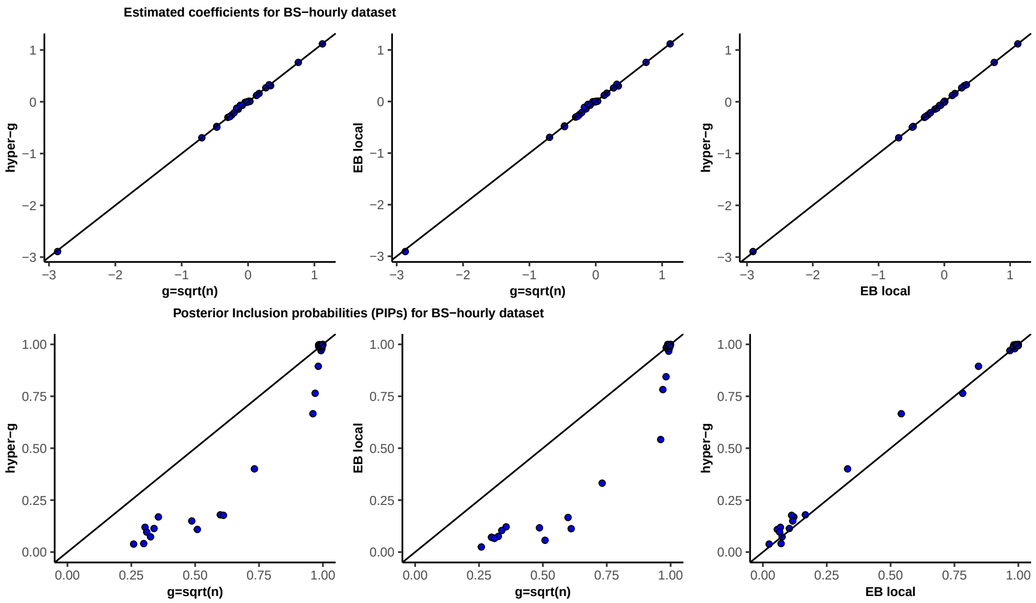
Estimated coefficients for BS−hourly dataset
hyper−g
EB local
g=sqrt(n)
EB local
Posterior Inclusion probabilities (PIPs) for BS−hourly dataset

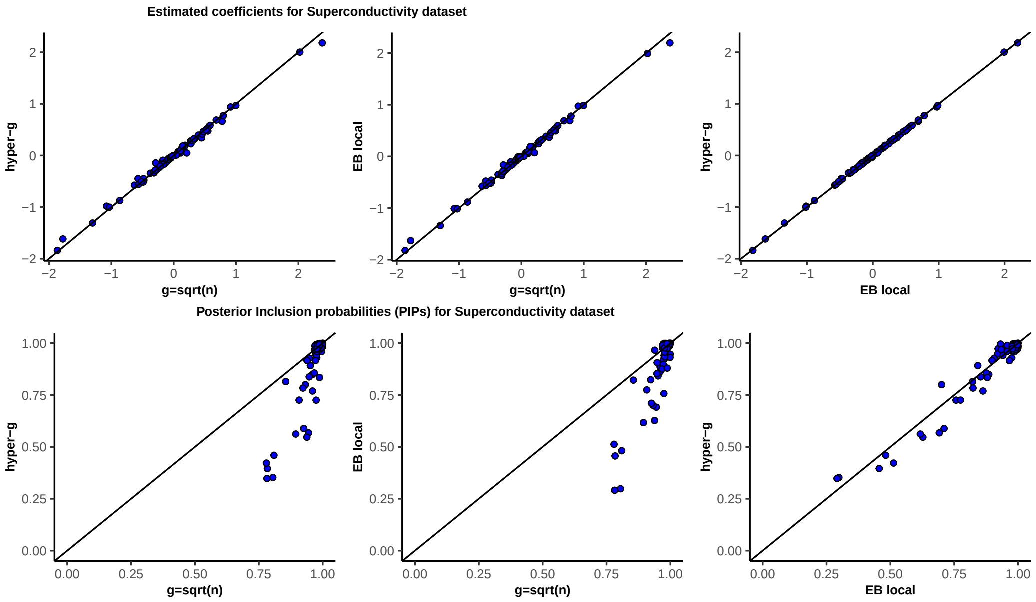
Estimated coefficients for Superconductivity dataset
hyper−g
EB local
g=sqrt(n)
EB local
Posterior Inclusion probabilities (PIPs) for Superconductivity dataset

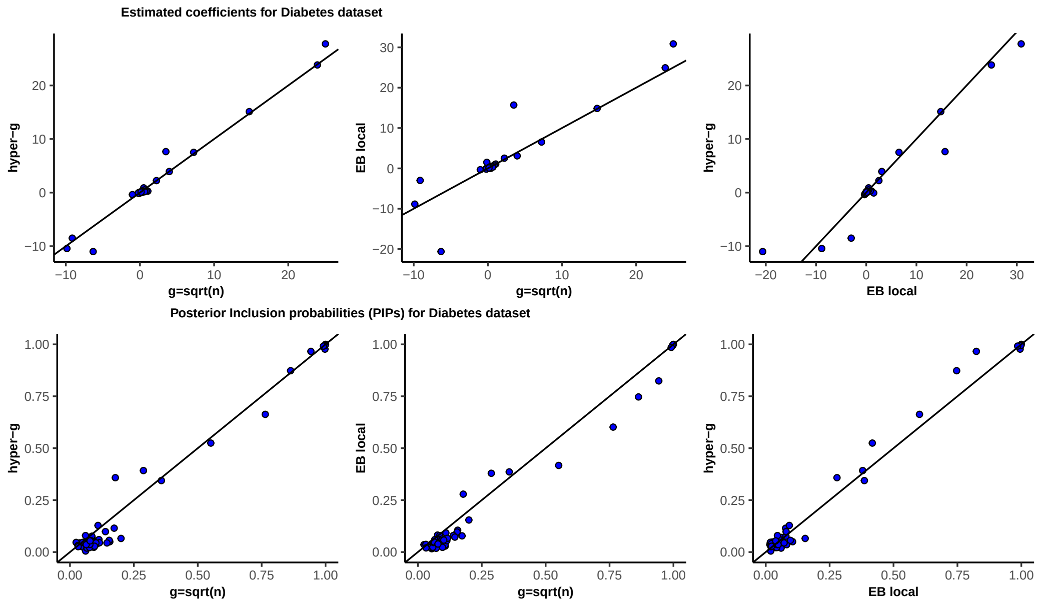
Estimated coefficients for Diabetes dataset
hyper−g
EB local
g=sqrt(n)
EB local
Posterior Inclusion probabilities (PIPs) for Diabetes dataset
hyper−g
EB local
g=sqrt(n)
EB local

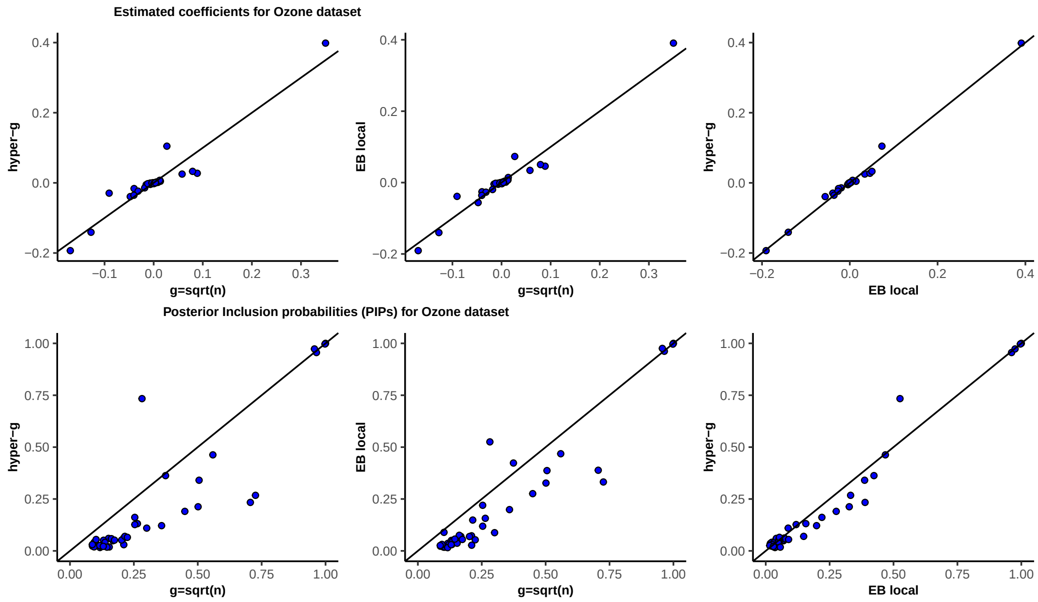
Estimated coefficients for Ozone dataset
hyper−g
EB local
g=sqrt(n)
EB local
Posterior Inclusion probabilities (PIPs) for Ozone dataset
hyper−g
EB local
g=sqrt(n)
EB local

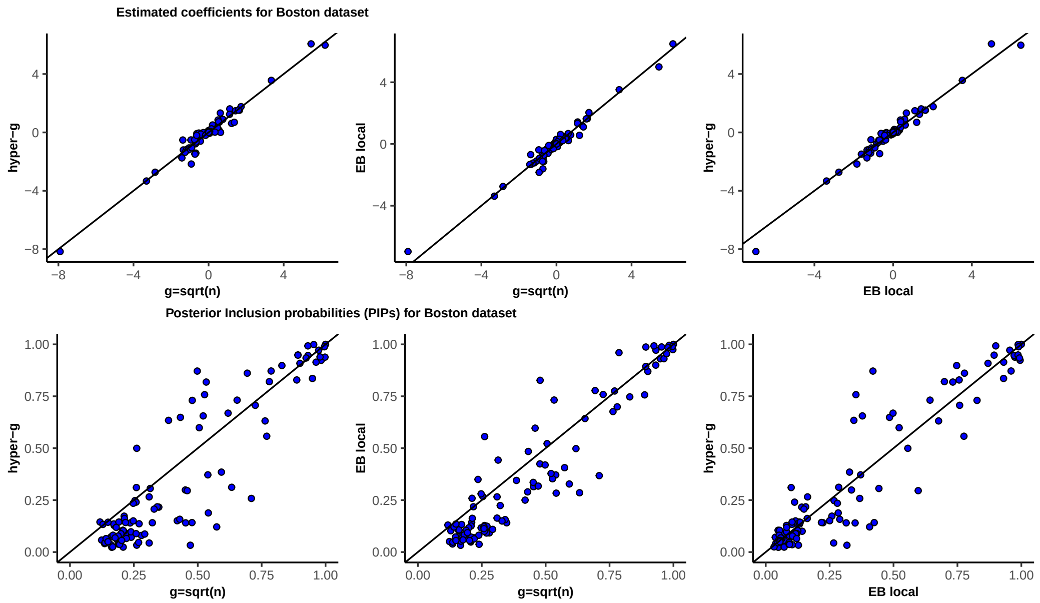
Estimated coefficients for Boston dataset
Posterior Inclusion probabilities (PIPs) for Boston dataset
hyper−g
EB local
g=sqrt(n)
EB local

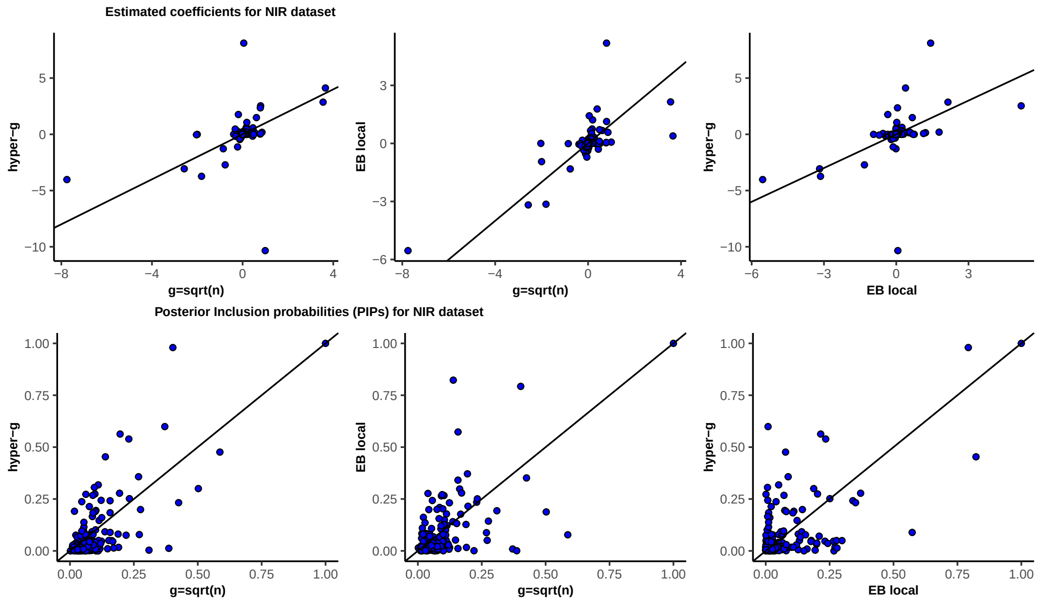
Estimated coefficients for NIR dataset
Posterior Inclusion probabilities (PIPs) for NIR dataset
hyper−g
EB local
g=sqrt(n)
EB local

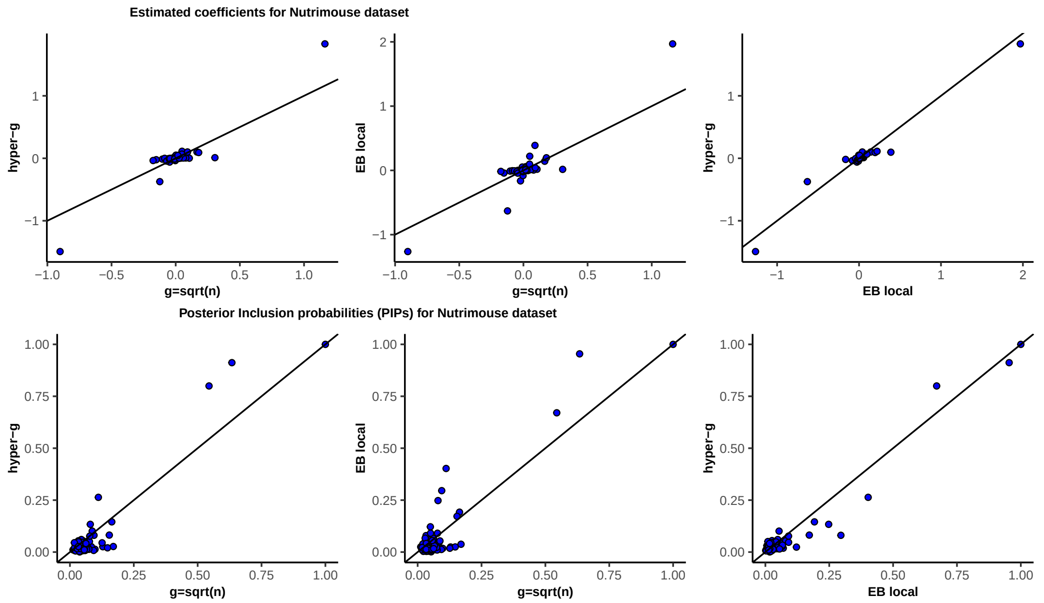
Estimated coefficients for Nutrimouse dataset
hyper−g
EB local
g=sqrt(n)
EB local
Posterior Inclusion probabilities (PIPs) for Nutrimouse dataset
hyper−g
EB local
g=sqrt(n)
EB local

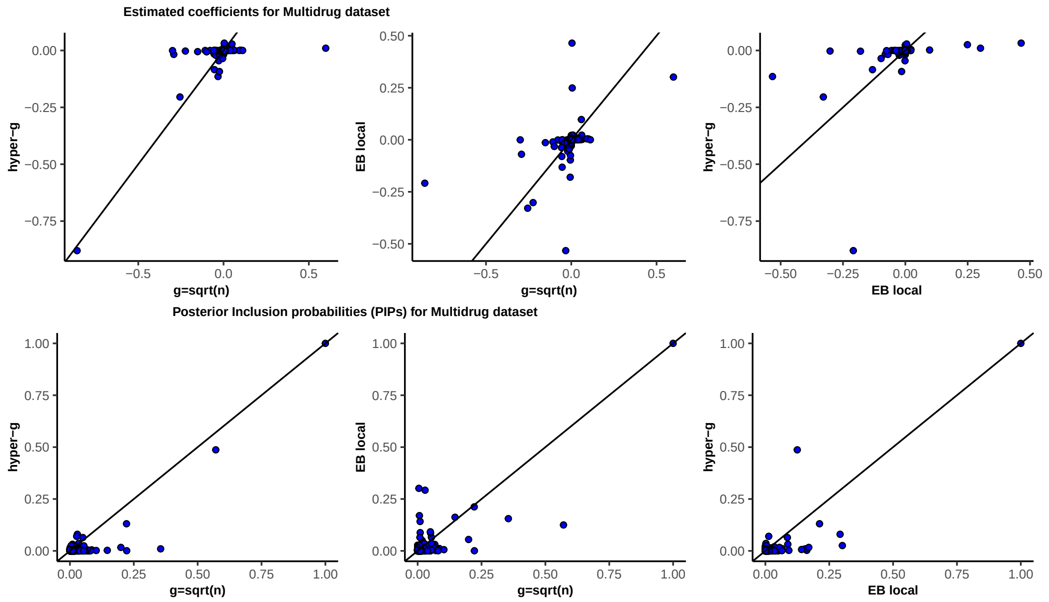
Estimated coefficients for Multidrug dataset
hyper−g
EB local
g=sqrt(n)
EB local
Posterior Inclusion probabilities (PIPs) for Multidrug dataset
hyper−g
EB local
g=sqrt(n)
EB local

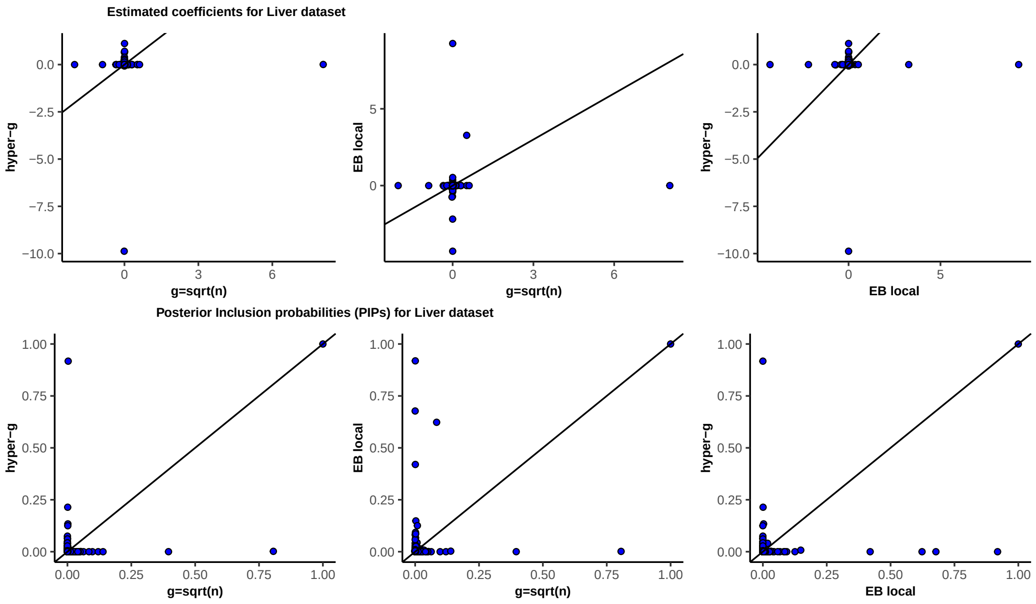
Estimated coefficients for Liver dataset
hyper−g
g=sqrt(n)
EB local
g=sqrt(n)
hyper−g
EB local
Posterior Inclusion probabilities (PIPs) for Liver dataset
hyper−g
g=sqrt(n)
EB local
g=sqrt(n)
hyper−g
EB local

3 Dataset specific results for all metrics from Table 1 of paper

	College	Tmax-Bias Correction	Tmin-Bias Correction	Bike Sharing Daily	Bike Sharing Hourly	SML 2010	Diabetes	Superconducitivity	Ozone	Boston Housing	Nutrimouse	multidrug	NIR	Liver
$g = \sqrt{n}$	22.227 (7.245)	2.537 (0.705)	2.226 (0.46)	1034.446 (298.497)	34.805 (9.633)	77.082 (21.875)	1687.135 (451.607)	24.079 (6.611)	20.003 (9.14)	616.984 (232.253)	273.596 (16.804)	114.398 (8.609)	529.441 (288.16)	580.114 (23.619)
Hyper-g	22.586 (7.721)	2.551 (0.768)	2.625 (0.58)	956.709 (267.792)	36.598 (9.394)	85.687 (27.862)	1759.479 (583.806)	24.356 (7.092)	18.695 (9.885)	632.621 (238.504)	236.12 (35.997)	116.132 (11.514)	618.573 (385.086)	592.795 (33.797)
EB-local	22.545 (7.621)	2.557 (0.776)	2.629 (0.578)	957.555 (272.537)	36.649 (9.496)	85.558 (28)	1747.042 (575.786)	23.984 (5.97)	18.871 (9.964)	636.934 (241.049)	236.609 (35.172)	115.641 (13.319)	589.34 (299.583)	594.547 (28.683)
JZS	22.794 (7.656)	2.569 (0.783)	2.667 (0.59)	957.291 (270.3)	37.391 (9.869)	86.346 (27.435)	1764.418 (498.718)	23.698 (5.913)	19.202 (9.753)	635.859 (230.23)	244.999 (43.769)	119.442 (12.467)	553.072 (339.772)	592.573 (32.734)
Horseshoe	18.981 (5.038)	2.558 (0.722)	2.205 (0.527)	1077.88 (269.534)	39.9 (10.175)	77.353 (22.17)	1606.644 (306.906)	31.088 (4.986)	20.421 (7.797)	645.507 (112.005)	227.22 (31.935)	114.84 (13.471)	292.609 (73.925)	609.424 (51.451)
UIP	22.476 (7.659)	2.651 (0.818)	2.884 (0.617)	978.286 (274.767)	40.422 (9.93)	86.912 (27.717)	1788.751 (381.509)	27.207 (9.039)	18.973 (9.816)	728.504 (230.12)	237.289 (32.48)	118.724 (13.716)	557.655 (314.593)	591.81 (31.976)
EB-global	22.726 (7.71)	2.549 (0.771)	2.633 (0.591)	951.792 (279.081)	36.33 (9.442)	86.094 (28.139)	1819.712 (728.26)	22.314 (5.894)	18.806 (10.462)	714.041 (316.618)	260.116 (41.044)	122.481 (11.471)	674.471 (500.263)	603.453 (37.125)
Benchmark	22.598 (7.623)	2.655 (0.814)	2.878 (0.609)	979.953 (272.206)	40.904 (10.893)	87.01 (27.811)	1926.384 (429.822)	25.354 (9.86)	19.68 (9.939)	844.045 (224.59)	298.62 (44.423)	119.506 (6.163)	586.319 (383.109)	586.448 (26.409)
NLP	27.621 (8.958)	3.289 (0.964)	4.143 (0.528)	1478.751 (411.05)	43.159 (8.815)	80.118 (27.627)	2226.935 (993.586)	27.833 (16.504)	20.422 (10.555)	594.975 (148.281)	240.53 (33.305)	131.037 (23.351)	324.223 (24.929)	569.775 (36.2)
LASSO	21.012 (7.077)	2.636 (0.741)	2.214 (0.593)	1278.31 (385.763)	45.321 (13.169)	77.527 (22.319)	1553.861 (389.273)	43.975 (6.013)	21.347 (7.567)	737.581 (77.445)	256.163 (31.322)	107.926 (7.468)	253.289 (45.72)	579.167 (6.186)
SCAD	24.506 (10.092)	2.517 (0.711)	2.121 (0.49)	1529.596 (568.747)	41.786 (16.041)	83.61 (30.896)	1739.529 (494.73)	40.783 (6.677)	23.485 (12.932)	878.922 (109.591)	299.729 (68.661)	113.935 (11.363)	384.369 (41.925)	590.617 (19.728)
BIC-BAS	22.494 (7.604)	2.646 (0.8)	2.885 (0.609)	972.276 (271.268)	40.549 (11.248)	86.579 (27.942)	1787.937 (375.556)	28.104 (12.947)	19.224 (10.07)	707.524 (263.783)	558.517 (298.812)	127.602 (20.128)	634.026 (405.168)	599.152 (30.036)
BICREG-SIS	22.161 (7.761)	2.822 (0.883)	3.311 (0.562)	980.928 (272.486)	41.712 (10.311)	96.856 (27.552)	1700.149 (483.911)	56.813 (10.349)	30.084 (14.05)	758.83 (196.946)	302.505 (93.614)	140.1 (24.626)	472.652 (161.101)	513.219 (133.737)
Spikeslab	31.239 (7.861)	3.836 (1.253)	4.189 (0.46)	1623.362 (375.367)	62.433 (20.77)	138.361 (23.25)	2182.946 (364.524)	37.74 (13.209)	22.325 (10.339)	975.739 (220.227)	291.055 (34.78)	120.872 (9.248)	467.337 (189.504)	588.711 (45.202)
Elastic net	22.366 (7.616)	2.646 (0.713)	2.215 (0.557)	1338.567 (433.078)	43.148 (12.819)	74.776 (20.983)	1585.461 (395.18)	57.759 (7.87)	23.52 (8.681)	726.937 (72.811)	260.475 (29.084)	107.066 (6.03)	266.535 (23.26)	576.978 (3.694)
MCP	24.511 (10.457)	2.522 (0.739)	2.132 (0.508)	1204.207 (488.497)	40.207 (13.934)	86.5 (32.141)	1768.974 (503.851)	42.068 (8.602)	29.733 (12.781)	900.889 (118.698)	341.086 (76.503)	120.677 (13.14)	390.171 (43.232)	610.649 (10.71)
SS Lasso	33.331 (8.237)	3.909 (1.425)	4.459 (0.39)	1531.5 (377.188)	50.695 (9.345)	123.306 (20.843)	2309.326 (165.478)	44.571 (12.755)	21.425 (11.731)	756.073 (106.423)	279.246 (24.555)	137.111 (19.451)	383.014 (93.883)	594.029 (16.983)
LASSO-1se	44.082 (5.856)	8.022 (1.141)	6.951 (0.967)	2605.602 (305.118)	111.284 (7.785)	158.858 (20.793)	1766.498 (267.723)	100.979 (11.861)	27.973 (6.491)	870.685 (44.102)	278.472 (31.503)	110.447 (6.027)	280.294 (42.131)	577.01 (4.227)
EMVS	26.166 (4.174)	2.643 (0.593)	2.418 (0.619)	1212.878 (482.105)	62.759 (14.277)	130.979 (7.863)	13454.014 (2456.259)	48.824 (4.566)	28.987 (6.895)	751.219 (211.286)	291.633 (13.067)	120.745 (11.997)	397.282 (32.126)	595.787 (5.419)
AIC	23.98 (8.102)	2.558 (0.69)	2.104 (0.457)	1293.306 (431.048)	42.407 (12.324)	73.978 (21.287)	11652.227 (66671.38)	59.213 (26.662)	30.316 (16.117)	2161.99 (4044.25)	831.263 (471.679)	225.118 (128.153)	33136.847 (61879.495)	628.459 (91.614)
$g = 1$	132.911 (3.471)	21.23 (0.44)	20.481 (0.256)	3277.732 (174.725)	309.368 (2.856)	437.009 (8.451)	7873.668 (13099.449)	133.435 (5.263)	44.624 (4.009)	842.916 (38.247)	319.35 (3.959)	115.955 (1.276)	386.166 (17.578)	574.697 (1.539)

Table S1: Average RMSE($\times$1000) for parameter estimates using various techniques for all datasets averaged over 100 bootstrapped samples; Numbers in brackets represent standard deviation over 100 bootstrapped samples

	College	Tmax-Bias Correction	Tmin-Bias Correction	Bike Sharing Daily	Bike Sharing Hourly	SML 2010	Diabetes	Superconducitivity	Ozone	Boston Housing	Nutrimouse	multidrug	NIR	Liver
$g = \sqrt{n}$	0.103 (0.041)	0.012 (0.003)	0.01 (0.002)	4.973 (2.017)	0.159 (0.042)	0.332 (0.082)	6.371 (2.472)	0.089 (0.018)	0.07 (0.036)	2.337 (0.88)	1.36 (0.389)	0.397 (0.063)	1.141 (0.371)	0.956 (0.099)
Hyper-g	0.117 (0.081)	0.012 (0.004)	0.014 (0.006)	4.447 (2.588)	0.179 (0.09)	0.415 (0.291)	6.785 (3.589)	0.095 (0.049)	0.06 (0.068)	3.04 (1.843)	1.195 (0.518)	0.409 (0.075)	1.401 (1.172)	0.985 (0.111)
EB-local	0.113 (0.079)	0.012 (0.004)	0.014 (0.006)	4.503 (2.728)	0.182 (0.097)	0.421 (0.296)	6.805 (3.747)	0.096 (0.046)	0.057 (0.069)	2.959 (1.737)	1.151 (0.49)	0.397 (0.078)	1.269 (0.655)	0.987 (0.117)
JZS	0.116 (0.081)	0.012 (0.005)	0.014 (0.007)	4.501 (2.62)	0.186 (0.1)	0.411 (0.294)	7.13 (4.058)	0.087 (0.033)	0.062 (0.071)	2.937 (1.671)	1.258 (0.522)	0.432 (0.082)	1.323 (0.784)	0.976 (0.114)
Horseshoe	0.092 (0.021)	0.012 (0.004)	0.01 (0.003)	4.9 (1.902)	0.179 (0.056)	0.342 (0.127)	8.721 (1.279)	0.14 (0.021)	0.101 (0.027)	2.717 (0.892)	1.098 (0.143)	0.416 (0.088)	0.868 (0.197)	1.141 (0.241)
UIP	0.115 (0.079)	0.013 (0.006)	0.019 (0.01)	4.819 (3.211)	0.242 (0.129)	0.427 (0.306)	8.547 (4.719)	0.129 (0.099)	0.065 (0.082)	4.375 (2.143)	1.121 (0.431)	0.431 (0.086)	1.426 (1.057)	0.976 (0.114)
EB-global	0.12 (0.087)	0.012 (0.005)	0.014 (0.007)	4.559 (3.083)	0.186 (0.106)	0.433 (0.321)	8.762 (4.827)	0.107 (0.056)	0.075 (0.101)	5.677 (2.963)	1.87 (0.658)	0.497 (0.069)	2.543 (2.011)	1.057 (0.133)
Benchmark	0.119 (0.083)	0.013 (0.005)	0.019 (0.01)	4.867 (3.137)	0.243 (0.129)	0.43 (0.311)	10.836 (5.247)	0.127 (0.127)	0.081 (0.102)	7.755 (2.901)	2.546 (0.436)	0.533 (0.029)	1.957 (1.388)	0.976 (0.099)
NLP	0.195 (0.136)	0.025 (0.011)	0.073 (0.019)	7.449 (4.721)	0.312 (0.152)	0.334 (0.201)	12.268 (5.033)	0.12 (0.102)	0.076 (0.09)	3.175 (1.776)	1.271 (0.495)	0.464 (0.174)	1.429 (0.355)	0.821 (0.188)
BIC-BAS	0.113 (0.079)	0.013 (0.005)	0.019 (0.01)	4.797 (3.143)	0.241 (0.123)	0.433 (0.309)	8.531 (4.934)	0.146 (0.148)	0.064 (0.087)	4.117 (2.431)	1.918 (0.843)	0.45 (0.076)	1.565 (1.148)	1.009 (0.104)
BMA -bicreg	0.103 (0.064)	0.014 (0.006)	0.029 (0.013)	4.587 (3.164)	0.239 (0.129)	0.55 (0.431)	5.756 (3.519)	0.798 (0.25)	0.282 (0.195)	6.894 (2.591)	2.477 (1.225)	0.507 (0.136)	2.859 (1.183)	0.614 (0.328)
Spikeslab	0.273 (0.16)	0.034 (0.02)	0.082 (0.018)	16.851 (6.23)	0.7 (0.43)	1.679 (0.773)	15.265 (4.798)	0.285 (0.219)	0.1 (0.094)	10.525 (2.886)	2.445 (0.214)	0.484 (0.056)	1.379 (1.024)	0.939 (0.198)
AIC	0.111 (0.039)	0.012 (0.003)	0.009 (0.002)	5.916 (2.208)	0.194 (0.058)	0.329 (0.087)	26.489 (66.571)	0.259 (0.045)	0.16 (0.06)	5.848 (3.77)	4.906 (3.133)	0.524 (0.117)	60.811 (101.497)	1.04 (0.133)
$g = 1$	1.452 (0.07)	0.38 (0.009)	0.327 (0.007)	44.855 (4.177)	4.667 (0.141)	7.163 (0.262)	55.205 (58.045)	1.491 (0.059)	0.32 (0.047)	5.46 (0.527)	1.959 (0.299)	0.497 (0.043)	1.959 (0.315)	0.979 (0.038)

Table S2: Average parameter Mean Interval Score using various techniques for all datasets averaged over 100 bootstrapped samples; Numbers in brackets represent standard deviation over 100 bootstrapped samples

	College	Tmax-Bias Correction	Tmin-Bias Correction	Bike Sharing Daily	Bike Sharing Hourly	SML 2010	Diabetes	Superconducitivity	Ozone	Boston Housing	Nutrimouse	multidrug	NIR	Liver
$g = \sqrt{n}$	0.033 (0.034)	0.002 (0.003)	0.002 (0.003)	0.017 (0.02)	0.008 (0.009)	0.004 (0.006)	0.162 (0.106)	0.026 (0.015)	0.086 (0.113)	0.152 (0.075)	0.517 (0.091)	0.802 (0.097)	0.644 (0.163)	0.932 (0.094)
Hyper-g	0.034 (0.038)	0.001 (0.003)	0.003 (0.004)	0.016 (0.025)	0.01 (0.01)	0.005 (0.009)	0.166 (0.105)	0.029 (0.023)	0.094 (0.133)	0.182 (0.097)	0.397 (0.142)	0.806 (0.114)	0.653 (0.191)	0.939 (0.097)
EB-local	0.034 (0.038)	0.002 (0.003)	0.003 (0.004)	0.015 (0.016)	0.011 (0.012)	0.005 (0.009)	0.165 (0.104)	0.03 (0.023)	0.09 (0.121)	0.182 (0.088)	0.4 (0.13)	0.792 (0.132)	0.693 (0.167)	0.953 (0.089)
JZS	0.033 (0.038)	0.002 (0.003)	0.003 (0.004)	0.015 (0.017)	0.011 (0.01)	0.005 (0.008)	0.162 (0.106)	0.026 (0.016)	0.092 (0.115)	0.177 (0.084)	0.421 (0.153)	0.829 (0.111)	0.665 (0.187)	0.951 (0.091)
Horseshoe	0.03 (0.035)	0.002 (0.009)	0.002 (0.003)	0.023 (0.021)	0.016 (0.018)	0.006 (0.014)	0.137 (0.093)	0.052 (0.021)	0.088 (0.122)	0.185 (0.069)	0.398 (0.112)	0.764 (0.15)	0.495 (0.113)	0.953 (0.071)
UIP	0.035 (0.04)	0.002 (0.003)	0.003 (0.005)	0.015 (0.016)	0.013 (0.011)	0.005 (0.008)	0.172 (0.109)	0.043 (0.039)	0.096 (0.123)	0.236 (0.098)	0.397 (0.128)	0.809 (0.122)	0.68 (0.201)	0.947 (0.08)
EB-global	0.034 (0.037)	0.001 (0.003)	0.003 (0.004)	0.014 (0.018)	0.009 (0.009)	0.005 (0.008)	0.163 (0.105)	0.028 (0.022)	0.09 (0.125)	0.208 (0.092)	0.446 (0.145)	0.86 (0.089)	0.704 (0.186)	0.945 (0.081)
Benchmark	0.034 (0.037)	0.001 (0.003)	0.003 (0.005)	0.015 (0.018)	0.011 (0.01)	0.005 (0.008)	0.185 (0.105)	0.039 (0.044)	0.111 (0.138)	0.356 (0.112)	0.643 (0.125)	0.95 (0.059)	0.737 (0.191)	0.939 (0.091)
NLP	0.033 (0.038)	0.002 (0.003)	0.006 (0.007)	0.034 (0.025)	0.017 (0.009)	0.004 (0.007)	0.196 (0.092)	0.026 (0.021)	0.102 (0.109)	0.173 (0.082)	0.458 (0.111)	0.693 (0.104)	0.476 (0.059)	0.869 (0.117)
LASSO	0.132 (0.099)	0.031 (0.02)	0.006 (0.008)	0.224 (0.087)	0.082 (0.016)	0.137 (0.053)	0.148 (0.092)	0.411 (0.024)	0.154 (0.109)	0.321 (0.042)	0.703 (0.06)	0.756 (0.084)	0.75 (0.066)	0.938 (0.066)
SCAD	0.04 (0.05)	0.001 (0.003)	0.003 (0.007)	0.027 (0.021)	0.025 (0.02)	0.076 (0.045)	0.151 (0.105)	0.32 (0.042)	0.257 (0.168)	0.349 (0.05)	0.764 (0.078)	0.82 (0.093)	0.814 (0.055)	0.969 (0.035)
BIC-BAS	0.033 (0.037)	0.002 (0.003)	0.003 (0.005)	0.016 (0.019)	0.013 (0.011)	0.005 (0.009)	0.172 (0.108)	0.049 (0.053)	0.093 (0.12)	0.217 (0.109)	0.567 (0.189)	0.845 (0.104)	0.707 (0.179)	0.967 (0.068)
BICREG-SIS	0.033 (0.036)	0.001 (0.002)	0.004 (0.006)	0.014 (0.017)	0.014 (0.016)	0.006 (0.009)	0.165 (0.122)	0.27 (0.08)	0.396 (0.256)	0.29 (0.106)	0.649 (0.2)	0.806 (0.127)	0.772 (0.16)	0.625 (0.247)
Spikeslab	0.037 (0.043)	0.002 (0.004)	0.008 (0.008)	0.034 (0.021)	0.03 (0.024)	0.021 (0.025)	0.203 (0.092)	0.075 (0.063)	0.125 (0.115)	0.45 (0.091)	0.584 (0.1)	0.787 (0.067)	0.678 (0.165)	0.883 (0.144)
Elastic net	0.16 (0.098)	0.035 (0.019)	0.007 (0.009)	0.263 (0.066)	0.091 (0.023)	0.247 (0.074)	0.159 (0.09)	0.423 (0.024)	0.224 (0.133)	0.323 (0.04)	0.762 (0.061)	0.754 (0.077)	0.799 (0.06)	0.914 (0.088)
MCP	0.036 (0.047)	0.001 (0.002)	0.002 (0.003)	0.028 (0.023)	0.025 (0.019)	0.108 (0.056)	0.156 (0.114)	0.317 (0.042)	0.303 (0.184)	0.363 (0.051)	0.801 (0.078)	0.883 (0.081)	0.79 (0.049)	0.993 (0.019)
SS Lasso	0.054 (0.07)	0.001 (0.002)	0.005 (0.007)	0.05 (0.05)	0.018 (0.019)	0.02 (0.013)	0.347 (0.097)	0.292 (0.087)	0.124 (0.187)	0.322 (0.099)	0.456 (0.103)	0.79 (0.113)	0.678 (0.147)	0.965 (0.043)
EMVS	0.021 (0.025)	0.003 (0.004)	0.002 (0.004)	0.184 (0.091)	0.042 (0.021)	0.022 (0.01)	0.88 (0.01)	0.103 (0.021)	0.13 (0.085)	0.427 (0.098)	0.708 (0.058)	0.813 (0.095)	0.79 (0.063)	0.99 (0.006)
AIC	0.038 (0.039)	0.002 (0.007)	0.002 (0.003)	0.025 (0.029)	0.012 (0.013)	0.004 (0.008)	0.179 (0.114)	0.062 (0.025)	0.122 (0.13)	0.183 (0.076)	0.718 (0.166)	0.894 (0.105)	0.788 (0.16)	0.962 (0.063)
$g = 1$	0.034 (0.037)	0.002 (0.004)	0.003 (0.004)	0.039 (0.024)	0.015 (0.013)	0.011 (0.011)	0.2 (0.114)	0.09 (0.026)	0.13 (0.1)	0.277 (0.056)	0.738 (0.076)	0.827 (0.095)	0.742 (0.096)	0.943 (0.057)

Table S3: Average 1-AUPRC for variable selection using various techniques for all datasets averaged over 100 bootstrapped samples; Numbers in brackets represent standard deviation over 100 bootstrapped samples

	College	Tmax-Bias Correction	Tmin-Bias Correction	Bike Sharing Daily	Bike Sharing Hourly	SML 2010	Diabetes	Superconducitivity	Ozone	Boston Housing	Nutrimouse	multidrug	NIR	Liver
$g = \sqrt{n}$	0.14 (0.022)	0.221 (0.009)	0.163 (0.006)	0.177 (0.031)	0.277 (0.006)	0.073 (0.008)	0.506 (0.054)	0.226 (0.006)	0.282 (0.051)	0.16 (0.044)	0.38 (0.121)	0.889 (0.17)	0.149 (0.045)	0.518 (0.181)
Hyper-g	0.14 (0.024)	0.221 (0.009)	0.164 (0.006)	0.177 (0.031)	0.277 (0.006)	0.072 (0.008)	0.51 (0.057)	0.226 (0.006)	0.282 (0.051)	0.168 (0.048)	0.42 (0.199)	0.921 (0.2)	0.155 (0.059)	0.532 (0.213)
EB-local	0.14 (0.023)	0.221 (0.009)	0.164 (0.006)	0.177 (0.031)	0.277 (0.006)	0.072 (0.008)	0.509 (0.056)	0.226 (0.006)	0.283 (0.054)	0.167 (0.047)	0.438 (0.246)	0.926 (0.215)	0.154 (0.053)	0.535 (0.235)
JZS	0.14 (0.024)	0.221 (0.009)	0.164 (0.006)	0.178 (0.031)	0.277 (0.006)	0.073 (0.008)	0.514 (0.058)	0.226 (0.006)	0.283 (0.052)	0.167 (0.048)	0.411 (0.203)	0.948 (0.196)	0.154 (0.056)	0.546 (0.242)
Horseshoe	0.139 (0.023)	0.221 (0.009)	0.163 (0.006)	0.176 (0.031)	0.277 (0.006)	0.072 (0.008)	0.51 (0.057)	0.226 (0.006)	0.276 (0.045)	0.155 (0.046)	0.419 (0.195)	1.048 (0.366)	0.148 (0.04)	0.765 (0.383)
UIP	0.14 (0.023)	0.221 (0.009)	0.164 (0.006)	0.178 (0.031)	0.277 (0.006)	0.073 (0.008)	0.52 (0.059)	0.227 (0.006)	0.282 (0.049)	0.174 (0.05)	0.407 (0.209)	0.969 (0.291)	0.153 (0.045)	0.531 (0.214)
EB-global	0.14 (0.024)	0.221 (0.009)	0.164 (0.006)	0.178 (0.031)	0.277 (0.006)	0.073 (0.008)	0.516 (0.059)	0.226 (0.006)	0.288 (0.056)	0.187 (0.056)	0.415 (0.228)	0.947 (0.171)	0.175 (0.053)	0.607 (0.248)
Benchmark	0.14 (0.024)	0.221 (0.009)	0.164 (0.006)	0.179 (0.03)	0.277 (0.006)	0.073 (0.008)	0.533 (0.06)	0.227 (0.006)	0.282 (0.045)	0.193 (0.055)	0.445 (0.209)	1.006 (0.094)	0.198 (0.071)	0.673 (0.225)
NLP	0.141 (0.024)	0.221 (0.009)	0.165 (0.006)	0.177 (0.03)	0.277 (0.006)	0.073 (0.008)	0.536 (0.062)	0.227 (0.006)	0.288 (0.051)	0.185 (0.055)	0.373 (0.174)	1.193 (0.416)	0.195 (0.049)	0.574 (0.183)
LASSO	0.139 (0.022)	0.221 (0.009)	0.163 (0.006)	0.176 (0.031)	0.277 (0.006)	0.072 (0.008)	0.508 (0.053)	0.228 (0.006)	0.286 (0.049)	0.159 (0.046)	0.431 (0.208)	0.86 (0.195)	0.174 (0.04)	0.578 (0.171)
SCAD	0.14 (0.024)	0.221 (0.009)	0.163 (0.006)	0.177 (0.031)	0.277 (0.006)	0.072 (0.008)	0.508 (0.059)	0.229 (0.006)	0.302 (0.065)	0.185 (0.056)	0.428 (0.233)	0.893 (0.221)	0.253 (0.055)	0.624 (0.224)
BIC-BAS	0.14 (0.024)	0.221 (0.009)	0.164 (0.006)	0.178 (0.031)	0.277 (0.006)	0.073 (0.008)	0.52 (0.059)	0.227 (0.006)	0.284 (0.052)	0.174 (0.049)	2.06 (3.051)	1.052 (0.407)	0.155 (0.06)	0.538 (0.217)
BICREG-SIS	0.139 (0.024)	0.221 (0.009)	0.164 (0.006)	0.179 (0.031)	0.277 (0.006)	0.073 (0.008)	0.503 (0.055)	0.243 (0.006)	0.361 (0.07)	0.192 (0.053)	0.716 (0.53)	1.511 (0.539)	0.198 (0.058)	0.642 (0.262)
Spikeslab	0.143 (0.024)	0.222 (0.009)	0.165 (0.006)	0.194 (0.032)	0.278 (0.006)	0.074 (0.008)	0.543 (0.06)	0.23 (0.006)	0.286 (0.046)	0.211 (0.067)	0.451 (0.179)	0.978 (0.139)	0.172 (0.049)	0.596 (0.232)
Elastic Net	0.14 (0.023)	0.221 (0.009)	0.163 (0.006)	0.176 (0.03)	0.277 (0.006)	0.072 (0.008)	0.512 (0.056)	0.227 (0.006)	0.285 (0.051)	0.156 (0.045)	0.457 (0.229)	0.838 (0.223)	0.175 (0.04)	0.559 (0.174)
MCP	0.14 (0.024)	0.221 (0.009)	0.163 (0.006)	0.176 (0.031)	0.277 (0.006)	0.072 (0.008)	0.509 (0.062)	0.229 (0.006)	0.3 (0.067)	0.187 (0.061)	0.577 (0.29)	0.951 (0.231)	0.244 (0.063)	0.683 (0.284)
SS Lasso	0.143 (0.024)	0.222 (0.009)	0.166 (0.006)	0.189 (0.031)	0.278 (0.006)	0.073 (0.008)	0.57 (0.043)	0.228 (0.006)	0.295 (0.051)	0.197 (0.056)	0.46 (0.399)	1.413 (0.48)	0.193 (0.057)	0.772 (0.261)
LASSO-1se	0.151 (0.019)	0.225 (0.009)	0.167 (0.006)	0.197 (0.029)	0.28 (0.006)	0.076 (0.008)	0.535 (0.048)	0.231 (0.006)	0.303 (0.04)	0.186 (0.067)	0.424 (0.149)	0.946 (0.099)	0.21 (0.057)	0.629 (0.128)
EMVS	0.141 (0.023)	0.221 (0.009)	0.163 (0.006)	0.873 (0.136)	0.324 (0.009)	0.083 (0.009)	0.622 (0.086)	0.23 (0.006)	0.292 (0.04)	0.177 (0.053)	0.484 (0.308)	0.941 (0.227)	0.366 (0.077)	0.636 (0.201)
AIC	0.14 (0.023)	0.221 (0.009)	0.163 (0.006)	0.177 (0.032)	0.277 (0.006)	0.072 (0.008)	0.515 (0.063)	0.226 (0.006)	0.304 (0.07)	0.169 (0.048)	5.911 (16.127)	2.223 (1.856)	6.825 (14.002)	0.647 (0.343)
$g = 1$	0.355 (0.014)	0.415 (0.006)	0.372 (0.006)	0.379 (0.024)	0.458 (0.004)	0.303 (0.009)	0.624 (0.035)	0.42 (0.004)	0.448 (0.031)	0.361 (0.037)	0.54 (0.082)	0.93 (0.055)	0.371 (0.042)	0.723 (0.077)

Table S4: Average $1 - R^2_{test}$ for predictions using various techniques for all datasets averaged over 100 bootstrapped samples; Numbers in brackets represent standard deviation over 100 bootstrapped samples

	College	Tmax-Bias Correction	Tmin-Bias Correction	Bike Sharing Daily	Bike Sharing Hourly	SML 2010	Diabetes	Superconducitivity	Ozone	Boston Housing	Nutrimouse	multidrug	NIR	Liver
$g = \sqrt{n}$	2.179 (0.234)	0.713 (0.019)	0.546 (0.015)	38.002 (5.12)	5.231 (0.08)	3.602 (0.186)	247.793 (15.455)	2.97 (0.061)	1.993 (0.221)	20.316 (4.29)	11.705 (0.993)	16.757 (3.279)	3.75 (0.286)	50.295 (14.652)
Hyper-g	2.14 (0.268)	0.715 (0.02)	0.546 (0.016)	38.426 (5.62)	5.24 (0.082)	3.593 (0.286)	248.108 (16.003)	2.974 (0.063)	2.011 (0.25)	20.57 (4.897)	10.968 (2.041)	16.79 (3.159)	3.655 (0.804)	51.785 (17.85)
EB-local	2.141 (0.269)	0.715 (0.019)	0.546 (0.016)	38.45 (5.65)	5.24 (0.084)	3.598 (0.285)	248.693 (15.881)	2.975 (0.063)	2.01 (0.251)	20.535 (4.929)	11.628 (4.026)	17.474 (4.342)	3.687 (0.792)	51.399 (15.28)
JZS	2.143 (0.269)	0.715 (0.019)	0.546 (0.016)	38.457 (5.684)	5.24 (0.083)	3.601 (0.291)	248.934 (16.13)	2.977 (0.062)	2.016 (0.257)	20.451 (4.948)	11.388 (3.056)	17.315 (4.267)	3.648 (0.775)	53.562 (16.963)
Horseshoe	2.132 (0.268)	0.716 (0.02)	0.546 (0.016)	38.532 (5.75)	5.229 (0.082)	3.582 (0.289)	248.065 (16.767)	2.976 (0.063)	1.986 (0.242)	20.042 (4.891)	12.461 (4.638)	22.87 (14.105)	3.588 (0.708)	155.243 (91.875)
UIP	2.139 (0.269)	0.715 (0.019)	0.546 (0.016)	38.498 (5.739)	5.243 (0.083)	3.6 (0.283)	248.952 (15.804)	2.978 (0.063)	2.023 (0.26)	21.355 (5.177)	10.918 (2.632)	17.722 (4.814)	3.645 (0.684)	51.671 (17.637)
EB-global	2.139 (0.271)	0.715 (0.019)	0.546 (0.016)	38.681 (5.775)	5.24 (0.083)	3.603 (0.292)	248.977 (16.472)	2.978 (0.063)	2.045 (0.262)	23.215 (5.58)	13.262 (8.906)	17.065 (4.434)	4.185 (1.167)	60.925 (24.562)
Benchmark	2.143 (0.272)	0.715 (0.019)	0.546 (0.016)	38.592 (5.719)	5.241 (0.082)	3.6 (0.288)	250.164 (14.532)	2.977 (0.063)	2.037 (0.268)	22.871 (5.435)	11.278 (2.755)	16.377 (3.082)	4.095 (0.715)	58.899 (20.197)
NLP	2.154 (0.267)	0.715 (0.019)	0.548 (0.016)	38.467 (5.719)	5.241 (0.085)	3.579 (0.283)	251.78 (16.576)	2.978 (0.063)	2.033 (0.267)	22.942 (5.337)	10.865 (3.875)	29.404 (18.712)	4.101 (0.935)	60.889 (22.19)
BIC-BAS	2.139 (0.268)	0.715 (0.019)	0.546 (0.016)	38.572 (5.616)	5.242 (0.084)	3.6 (0.286)	249.039 (15.323)	2.977 (0.062)	2.023 (0.253)	21.249 (4.973)	42.039 (34.339)	18.389 (6.603)	3.706 (0.831)	53.948 (21.88)
BICREG-SIS	2.139 (0.268)	0.714 (0.019)	0.546 (0.016)	38.724 (5.711)	5.235 (0.082)	3.625 (0.301)	248.026 (17.193)	3.041 (0.058)	2.271 (0.253)	24.199 (5.976)	34.378 (22.277)	54.369 (24.741)	4.323 (1.081)	90.871 (53.324)
Spikeslab	2.16 (0.27)	0.715 (0.019)	0.549 (0.016)	39.647 (5.737)	5.231 (0.087)	3.697 (0.294)	251.747 (15.192)	2.984 (0.062)	2.063 (0.282)	24.519 (6.453)	11.586 (3.199)	16.638 (3.788)	3.82 (0.895)	57.489 (23.42)
AIC	2.144 (0.268)	0.716 (0.019)	0.545 (0.016)	38.481 (5.804)	5.23 (0.081)	3.565 (0.292)	256.391 (21.61)	2.976 (0.062)	2.037 (0.261)	18.909 (3.655)	89.41 (48.949)	26.811 (14.668)	7.875 (2.61)	60.016 (25.634)
$g = 1$	3.278 (0.07)	0.923 (0.009)	0.861 (0.015)	51.261 (2.987)	6.435 (0.016)	7.356 (0.063)	273.295 (6.171)	3.781 (0.014)	2.574 (0.147)	32.661 (2.993)	14.934 (1.201)	15.953 (1.415)	5.897 (0.108)	60.632 (11.203)

Table S5: Average Mean Interval Score for predictions using various techniques for all datasets averaged over 100 bootstrapped samples; Numbers in brackets represent standard deviation over 100 bootstrapped samples

	College	Tmax-Bias Correction	Tmin-Bias Correction	Bike Sharing Daily	Bike Sharing Hourly	SML 2010	Diabetes	Superconducitivity	Ozone	Boston Housing	Nutrimouse	multidrug	NIR	Liver
$g=\sqrt{n}$	9.07 (0.42)	19.78 (0.31)	21.06 (0.35)	21.8 (0.97)	25.34 (1.02)	19.56 (0.5)	11.26 (1.56)	77.37 (0.85)	12.13 (1.32)	38.55 (3.18)	8.12 (1.53)	6.04 (3.05)	16.2 (1.74)	5.13 (1.46)
Hyper-g	6.79 (0.34)	17.79 (0.42)	17.3 (1.55)	17.64 (1)	20.66 (1.03)	15.93 (0.68)	8.96 (1.69)	71.27 (1.9)	8.48 (1.12)	31.47 (3.22)	7.19 (1.92)	5.68 (3.48)	13.88 (1.6)	4.52 (1.28)
EB-local	6.8 (0.32)	17.79 (0.42)	17.3 (1.48)	17.66 (1.02)	20.67 (1.02)	15.92 (0.66)	9.18 (1.72)	71.23 (1.78)	8.47 (1.14)	31.67 (3.4)	7.63 (2.04)	6.37 (2.9)	13.72 (1.64)	4.51 (1.22)
JZS	6.72 (0.32)	17.69 (0.44)	16.94 (1.57)	17.31 (1.03)	20.28 (0.99)	15.87 (0.66)	7.37 (1.2)	70.05 (2.07)	7.94 (1.06)	30.85 (3.28)	6.62 (2.01)	3.65 (2.39)	13.64 (1.71)	4.33 (1.2)
Horseshoe	8.64 (1.05)	17.82 (0.77)	20.8 (0.65)	21.4 (1.33)	27.16 (1.38)	18.93 (0.97)	8.02 (1.59)	73.52 (1.62)	12.3 (2)	41.84 (3.85)	5.29 (3.24)	2.24 (2.65)	3.2 (1.24)	8.59 (4.81)
UIP	6.81 (0.32)	17.11 (0.46)	15.25 (1.29)	15.97 (0.95)	18.92 (0.73)	15.78 (0.62)	5.88 (0.86)	61.39 (1.39)	7.29 (0.82)	26.33 (2.74)	6.88 (1.85)	3.6 (1.85)	13.15 (1.66)	4.69 (1.35)
EB-global	6.71 (0.33)	17.85 (0.43)	17.53 (1.67)	16.81 (1.08)	20.41 (1.09)	15.9 (0.69)	6.48 (1.1)	74.72 (3.42)	6.75 (0.87)	27.62 (3.96)	3.72 (0.83)	1.76 (0.38)	10.57 (1.61)	3.13 (0.73)
Benchmark	6.82 (0.32)	17.11 (0.47)	15.31 (1.3)	15.58 (0.89)	18.93 (0.73)	15.79 (0.63)	4.21 (0.53)	61.24 (1.42)	5.85 (0.52)	15.71 (2.38)	2.62 (0.39)	1.09 (0.2)	6.66 (0.99)	2.06 (0.32)
NLP	4.57 (0.32)	14.06 (0.43)	9.57 (0.59)	18.28 (1.1)	17.89 (0.5)	16.05 (0.85)	3 (0.62)	66.23 (1.42)	5.1 (0.82)	28.3 (4.08)	20.01 (2.86)	32.74 (3.25)	35.04 (2.39)	28.19 (5.09)
LASSO	12.2 (1.62)	20.83 (1.01)	21.83 (0.38)	26.77 (1.43)	31.38 (1.15)	21.31 (1.57)	18.23 (4.82)	79.51 (1.48)	17.52 (4.29)	67.29 (11.57)	15.75 (7.41)	19.48 (12.21)	36.46 (3.31)	24.4 (12.81)
SCAD	10.15 (2.19)	18.51 (0.73)	21.73 (0.45)	23.24 (3.31)	29.43 (1.41)	17.63 (1.87)	15.35 (3.04)	62.58 (1.58)	12.04 (3.26)	48.89 (17.74)	7.02 (2.44)	9.64 (4.45)	11.59 (1.9)	11.66 (3.87)
BIC-BAS	6.84 (0.32)	17.12 (0.47)	15.31 (1.3)	16.07 (0.95)	18.91 (0.71)	15.86 (0.66)	5.95 (0.89)	61.25 (1.32)	7.32 (0.86)	27.08 (3.01)	25.76 (2.57)	6.64 (6.09)	14.05 (1.72)	5.2 (1.41)
BICREG-SIS	6.99 (0.27)	16.48 (0.46)	13.73 (0.72)	15.44 (0.78)	18.56 (0.59)	14.95 (0.48)	8.33 (0.61)	29.9 (0.32)	3.29 (1.24)	23.94 (2.24)	7.68 (0.91)	10.4 (1.09)	9 (1.18)	8.27 (1.64)
Spikeslab	4.03 (0.34)	13.4 (0.31)	9.06 (0.49)	10.14 (0.6)	15.81 (0.9)	12.35 (0.62)	2.22 (0.19)	45.35 (2.04)	3.94 (0.18)	8.91 (1.38)	1.55 (0.35)	0.35 (0.24)	5.66 (0.39)	1.84 (0.47)
Elastic Net	13.49 (1.57)	20.94 (1.04)	21.84 (0.37)	26.88 (1.48)	31.62 (1.05)	22.06 (0.98)	20.24 (6.26)	80.27 (0.95)	20.53 (4.75)	76.05 (9.84)	26.94 (14.26)	51.66 (35.9)	47.06 (10)	60.37 (39.08)
MCP	9.31 (2.39)	18.26 (0.61)	21.74 (0.44)	23.58 (3.6)	29.17 (1.58)	17.54 (1.94)	11.37 (2.36)	62.64 (1.46)	10.49 (3.1)	47.92 (18.13)	5.3 (2.15)	4.27 (2.17)	11.97 (2.16)	6.08 (2.41)
SS Lasso	5.25 (0.48)	14.27 (0.45)	9.4 (0.64)	12.1 (1.09)	16.48 (0.69)	13.48 (0.67)	3 (0)	66.43 (1.78)	5.04 (0.55)	17.9 (2.49)	3.55 (1.01)	5.89 (2.81)	7.6 (0.79)	2.56 (0.83)
LASSO-1se	8.9 (0.81)	18.66 (0.76)	16.81 (1.32)	17.8 (2.04)	18.48 (1.84)	17.6 (1.01)	8.62 (2.35)	67.56 (1.8)	10.39 (1.75)	41.52 (11.98)	9.12 (4.61)	5.43 (6.62)	29.65 (5.13)	10.27 (6.73)
EMVS	1 (0)	1 (0)	1 (0)	18.63 (1.44)	7.53 (0.72)	8.25 (0.69)	56.58 (2.37)	36.45 (1.43)	1.69 (0.51)	40.11 (3.95)	5.56 (1.48)	0.34 (0.59)	3.09 (1.13)	5.46 (2.95)
AIC	12.72 (0.46)	21.31 (0.1)	21.72 (0.07)	26.46 (0.45)	31.55 (0.28)	21.9 (0.18)	22.85 (3.69)	80.15 (0.21)	22.35 (3.27)	77.2 (5.82)	27.85 (0.49)	26.86 (8.92)	95.85 (12.23)	14.79 (8.66)
$g=1$	11.56 (0.4)	21.34 (0.08)	20.82 (0.19)	25.7 (0.52)	31.58 (0.27)	20.74 (0.33)	30.97 (5.26)	79.69 (0.28)	21.07 (2.06)	41.5 (3.99)	10.15 (2.14)	5.11 (2.92)	20.29 (2.81)	4.24 (1.59)

Table S6: Average model size for predictions using various techniques for all datasets averaged over 100 bootstrapped samples; Numbers in brackets represent standard deviation over 100 bootstrapped samples

	College	Tmax-Bias Correction	Tmin-Bias Correction	Bike Sharing Daily	Bike Sharing Hourly	SML 2010	Diabetes	Superconducitivity	Ozone	Boston Housing	Nutrimouse	multidrug	NIR	Liver
$g = \sqrt{n}$	0.055 (0.003)	0.166 (0.01)	0.15 (0.008)	0.201 (0.009)	0.288 (0.051)	0.15 (0.007)	0.191 (0.027)	0.626 (0.133)	0.144 (0.016)	0.363 (0.107)	0.185 (0.018)	0.389 (0.076)	0.277 (0.024)	2.306 (0.663)
Hyper-g	0.481 (0.092)	1.214 (0.073)	1.117 (0.045)	1.29 (0.115)	2.434 (0.139)	0.466 (0.019)	0.307 (0.033)	3.135 (0.207)	0.34 (0.036)	0.982 (0.138)	0.36 (0.099)	0.436 (0.096)	0.538 (0.048)	2.34 (0.647)
EB-local	0.045 (0.003)	0.156 (0.01)	0.148 (0.008)	0.154 (0.013)	0.242 (0.061)	0.134 (0.009)	0.167 (0.024)	0.567 (0.112)	0.111 (0.011)	0.261 (0.075)	0.174 (0.023)	0.385 (0.079)	0.23 (0.016)	2.189 (0.608)
JZS	1.833 (0.154)	3.24 (0.195)	2.881 (0.116)	1.478 (0.065)	3.374 (0.86)	2.251 (0.102)	1.892 (0.131)	5.207 (1.763)	1.637 (0.104)	1.415 (0.308)	1.822 (0.098)	1.409 (0.231)	1.881 (0.065)	5.781 (3.093)
Horseshoe	3 (0.089)	6.857 (0.453)	6.558 (0.161)	3.492 (0.059)	16.174 (1.275)	3.716 (0.092)	7.627 (0.376)	32.715 (8.086)	5.351 (0.183)	8.558 (1.741)	3.966 (0.082)	10.246 (1.57)	18.304 (0.518)	187.127 (130.548)
UIP	0.044 (0.003)	0.155 (0.053)	0.144 (0.031)	0.141 (0.056)	0.22 (0.029)	0.134 (0.025)	0.139 (0.035)	0.413 (0.087)	0.109 (0.03)	0.219 (0.093)	0.179 (0.035)	0.348 (0.089)	0.224 (0.031)	2.204 (0.641)
EB-global	0.05 (0.004)	0.161 (0.01)	0.154 (0.008)	0.168 (0.015)	0.243 (0.018)	0.141 (0.008)	0.188 (0.026)	0.569 (0.11)	0.122 (0.013)	0.283 (0.079)	0.205 (0.029)	0.413 (0.101)	0.246 (0.019)	2.348 (0.705)
Benchmark	0.044 (0.003)	0.155 (0.03)	0.146 (0.033)	0.139 (0.032)	0.224 (0.021)	0.136 (0.027)	0.131 (0.042)	0.561 (0.295)	0.102 (0.035)	0.16 (0.042)	0.122 (0.006)	0.3 (0.06)	0.194 (0.01)	2.132 (0.611)
NLP	0.037 (0.005)	0.671 (0.08)	0.343 (0.067)	1.01 (0.174)	1.325 (0.186)	0.734 (0.105)	1.02 (0.66)	7.537 (2.069)	0.706 (0.187)	6.963 (2.985)	27.281 (11.043)	407.185 (181.362)	75.628 (19.532)	1044.758 (828.306)
LASSO	0.11 (0.009)	0.237 (0.019)	0.232 (0.009)	0.12 (0.008)	0.49 (0.026)	0.14 (0.007)	0.485 (0.13)	1.434 (0.16)	0.23 (0.036)	0.478 (0.157)	0.168 (0.013)	0.178 (0.032)	0.438 (0.081)	1.172 (1.062)
SCAD	0.155 (0.011)	1.525 (0.108)	1.458 (0.081)	0.383 (0.029)	6.449 (0.464)	0.589 (0.054)	2.936 (0.708)	61.655 (7.135)	0.798 (0.191)	4.109 (0.884)	0.098 (0.006)	0.361 (0.042)	0.221 (0.023)	3.821 (3.5)
BIC-BAS	0.044 (0.003)	0.152 (0.01)	0.144 (0.01)	0.141 (0.033)	0.227 (0.033)	0.133 (0.009)	0.136 (0.016)	0.539 (0.103)	0.104 (0.009)	0.221 (0.078)	0.474 (0.04)	0.373 (0.105)	0.223 (0.015)	2.155 (0.536)
BICREG-SIS	0.5 (0.05)	1.434 (0.102)	1.958 (0.489)	2.996 (0.181)	5.904 (1.39)	1.498 (0.059)	12.444 (2.161)	34.744 (4.794)	0.332 (0.075)	63.468 (32.842)	0.729 (0.138)	1.991 (0.551)	2.704 (0.56)	5.651 (2.524)
Spikeslab	0.359 (0.028)	1.398 (0.099)	0.937 (0.104)	1.482 (0.175)	2.561 (0.186)	1.387 (0.101)	1.496 (0.175)	9.037 (2.267)	1.211 (0.088)	4.055 (1.063)	5.487 (0.207)	21.35 (5.115)	12.186 (0.846)	113.032 (35.903)
Elastic net	6.775 (0.273)	14.366 (0.696)	17.329 (1.196)	10.89 (0.61)	30.132 (6.08)	9.925 (0.384)	20.376 (2.659)	68.895 (5.133)	10.484 (0.85)	19.929 (4.058)	10.138 (0.612)	14.569 (1.167)	24.1 (1.785)	59.483 (25.401)
MCP	0.15 (0.008)	1.435 (0.125)	1.447 (0.088)	0.318 (0.029)	5.443 (0.349)	0.545 (0.044)	2.964 (0.672)	59.008 (6.804)	0.792 (0.196)	4.279 (0.875)	0.09 (0.004)	0.273 (0.029)	0.176 (0.011)	0.817 (0.145)
SS Lasso	0.015 (0.006)	0.14 (0.028)	0.211 (0.05)	0.015 (0.004)	0.442 (0.105)	0.039 (0.007)	0.013 (0.002)	4.179 (0.917)	0.082 (0.015)	0.07 (0.013)	0.082 (0.023)	0.539 (0.129)	0.225 (0.041)	0.86 (0.866)
LASSO-1se	0.11 (0.009)	0.237 (0.019)	0.232 (0.009)	0.12 (0.008)	0.49 (0.026)	0.14 (0.007)	0.485 (0.13)	1.434 (0.16)	0.23 (0.036)	0.478 (0.157)	0.168 (0.013)	0.178 (0.032)	0.438 (0.081)	1.172 (1.062)
EMVS	0.046 (0.003)	0.134 (0.008)	0.131 (0.004)	0.439 (0.058)	0.348 (0.021)	0.071 (0.004)	8.293 (5.131)	2.731 (0.566)	0.348 (0.043)	3.016 (0.982)	0.465 (0.01)	0.771 (0.081)	9.176 (0.41)	4.204 (1.54)
AIC	0.056 (0.003)	0.168 (0.011)	0.153 (0.012)	0.19 (0.009)	0.298 (0.051)	0.151 (0.008)	0.356 (0.113)	1.365 (0.437)	0.246 (0.057)	1.055 (0.357)	0.525 (0.036)	0.638 (0.144)	2.021 (2.429)	2.344 (0.665)
$g = 1$	0.056 (0.004)	0.141 (0.009)	0.114 (0.007)	0.171 (0.012)	0.253 (0.053)	0.114 (0.007)	0.608 (0.147)	1.657 (0.364)	0.321 (0.047)	0.664 (0.236)	0.23 (0.028)	0.435 (0.095)	0.456 (0.069)	2.716 (0.837)

Table S7: Average computation using various techniques for all datasets averaged over 100 bootstrapped samples; Numbers in brackets represent standard deviation over 100 bootstrapped samples

4 R^2_{test} vs. $\hat{p}$ plots for all datasets

For tall datasets ($n > p$), we observe that Bayesian techniques select sparser models with similar/superior prediction accuracy. Figure 1 illustrates our claim for tall datasets. Note that $g = 1$ is excluded from the graphs since it had significantly lower R^2_{test} compared to other techniques and EMVS is excluded since it does not provide a sparse estimate in its default settings. In particular, we note that AIC, LASSO-λ_{min} and elastic net consistently tend to select denser models for each dataset without any increase in the prediction accuracy. We also note that while $LASSO = \lambda_{1se}$ selects sparser model, it tends to have lower accuracy than other Bayesian techniques at the same sparsity levels. We also note that Spike-Slab and SSLASSO tent to select very sparse models with slightly lower accuracy compared to other methods. Most other methods are clustered together in terms of both average model size and point predictive accuracy.

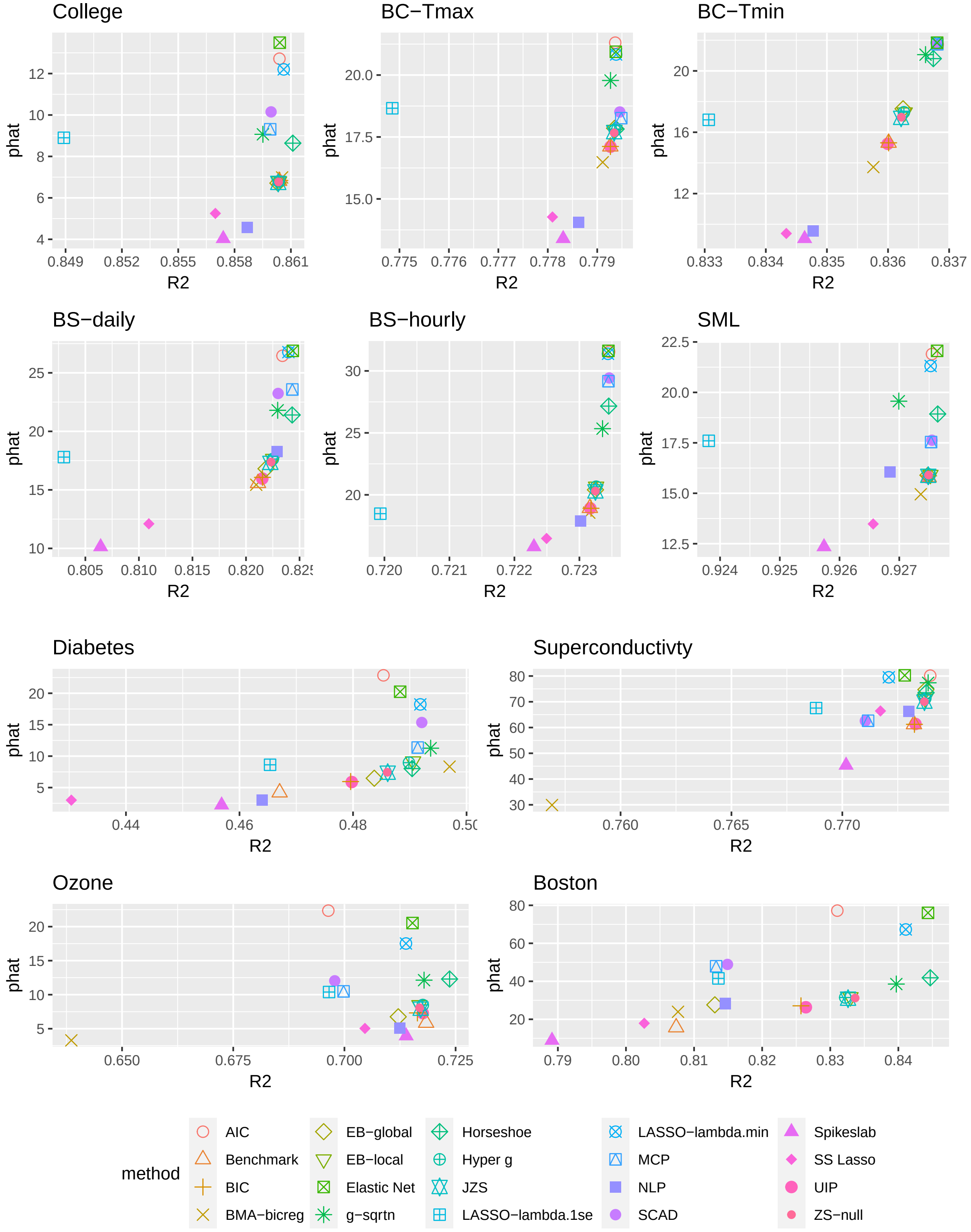

Figure 1: R^2_{test} vs. $\hat{p}$ plotted for all the tall datasets; $g = 1$ is excluded because it had much lower R^2 than other techniques in all the datasets

For wide datasets ($p > n$), we observe much more variability in the predictive accuracy along with average model size. We note that AIC, BIC and bicreg, tend to overfit models by selecting models with $p \approx n_{train}$ and often perform worse than baseline thus having negative R^2_{test}. We excluded $g = 1$ and EMVS for the same reason as tall datasets. We observe Elastic Net and LASSO-λ_{min} still tend to have much higher average model size without significant increase in accuracy with an exception in Multidrug dataset. Among Bayesian techniques, NLP tends to select denser models. We note that $g = \sqrt{n}$ tend to consistently outperform the other techniques in terms of superior accuracy with sparse models.

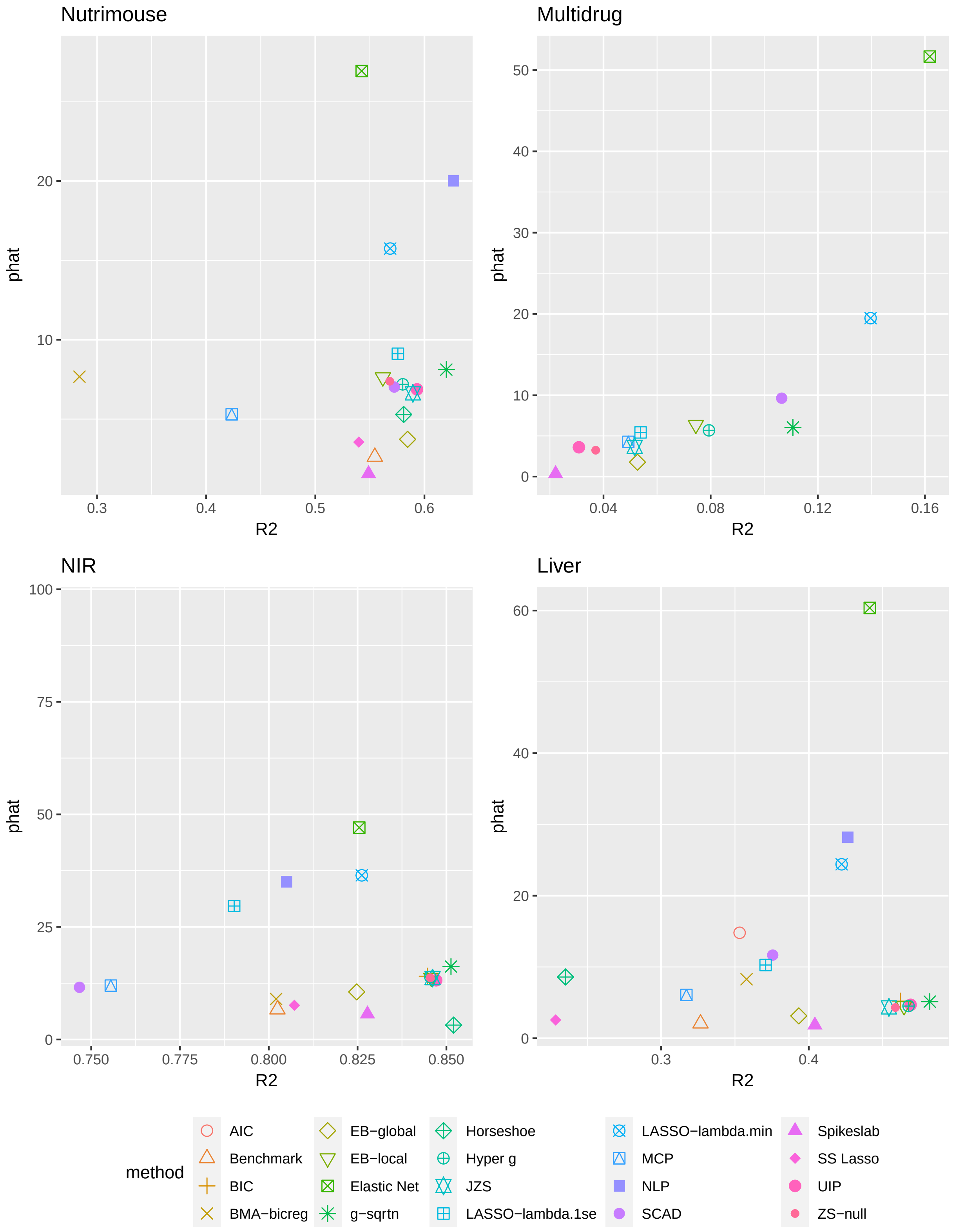

Figure 2: R^2_{test} vs. $\hat{p}$ plotted for all the wide datasets; Methods with negative R^2_{test} and $g = 1$ are excluded from the plot since they had significantly lower R^2 compared to other techniques in the study for all the wide datasets
